# Cilostazol-inhibited RhoA/NF-κB signaling mitigates hippocampal inflammation and post-stroke depression

**DOI:** 10.3389/fphar.2025.1643343

**Published:** 2025-09-03

**Authors:** Yuling Zhang, Yichen Li, Yafang Wu, Xiying Tan, Tingting Ji, Chaozhi Tang

**Affiliations:** ^1^ School of Biological Science and Technology, Liupanshui Normal University, Liupanshui, China; ^2^ College of Life Sciences, Henan Normal University, Xinxiang, China; ^3^ Department of Neurology, Xinxiang City First People’s Hospital, Xinxiang, China

**Keywords:** post-stroke depression, cilostazol, microglial activation, neuronal apoptosis, hippocampal inflammation, RhoA/NF-κB signaling

## Abstract

**Introduction:**

Although approximately one-third of stroke survivors develop post-stroke depression (PSD), clinically recommended preventive treatments currently are unavailable. Cilostazol, an established stroke therapeutic, has demonstrated potential in preventing PSD, however, its neuroprotective mechanisms remain unclarified. This study elucidates the molecular pathways via which cilostazol may protect against PSD development.

**Methods:**

Middle cerebral artery occlusion (MCAO) was performed on C57BL/6J mice to establish an ischemic stroke (IS) model. Subsequently, the IS mice were treated with cilostazol and subjected to chronic unpredictable mild stress (CUMS) to induce PSD. Cilostazol’s PSD prevention efficacy was evaluated using the sucrose preference, open field, tail suspension, and Morris water maze. Nissl staining and immunofluorescence labeling were used to detect cilostazol’s effects on hippocampal neuronal apoptosis and microglial activation. Western blot and quantitative polymerase chain reaction were used to investigate cilostazol’s regulation of hippocampal inflammation and apoptosis factors. Cilostazol’s potential PSD-preventive mechanism was further explored by examining the primary hippocampal neuronal apoptosis induced by RhoA-activated BV2 microglia.

**Results:**

Cilostazol intervention significantly suppressed hippocampal microglial proliferation and activation and decreased pro-inflammatory cytokine expression. These changes were associated with attenuated hippocampal neuronal swelling and apoptosis and were accompanied by apparent alleviation of depressive behaviors in CUMS-subjected IS mice. Mechanistically, *in vitro* experiments demonstrated that cilostazol inhibited RhoA/NF-κB signaling pathway activation in BV2 microglia, leading to decreased tumor necrosis factor-alpha and interleukin-1β secretion. The neuroprotective effects of cilostazol, potentially mediated via a cAMP-dependent reduction of microglia-induced neuronal damage, may contribute to the improvement of depressive-like behaviors in mice with PSD.

**Conclusion:**

Cilostazol may alleviate hippocampal inflammation by inhibiting RhoA/NF-κB signaling pathway activation in the microglia, providing neuronal protection and PSD prevention effects.

## 1 Introduction

Ischemic stroke (IS) is an acute cerebrovascular disorder, which can cause death when the vascular infarction is severe (Gan et al., 2025). Approximately one-third of patients with IS develop post-stroke depression (PSD) (Medeiros et al., 2020; Chun et al., 2022; Feng et al., 2024; Zhang et al., 2024c). Patients with PSD exhibit more severe clinical symptoms, including delayed consciousness, memory loss, language and cognitive impairments, sleep disturbances, emotional depression, difficulty cooperating with treatment, and a higher risk of suicidal tendencies, than those with simple IS. Consequently, their recovery rate is significantly reduced (Medeiros et al., 2020; Feng et al., 2024; Gao et al., 2025). Medications that regulate neural activities such as consciousness, cognition, or mood can inevitably produce some side effects; hence, patients with IS may experience stronger drug-related adverse reactions. Clinically, it is not recommended to administer additional antidepressant medications to patients with IS to prevent the occurrence of PSD. Consequently, PSD development has become a major obstacle to improving the recovery rate of these patients (2020b; 2020a).

Advanced research into the inflammatory mechanisms of depression have revealed that patients with PSD have high levels of inflammation (Tang et al., 2016; Wu et al., 2020; Wen et al., 2024; Zhou et al., 2025). The pathomechanism of inflammation, from IS to PSD, has recently garnered attention. Post-IS, the microglia and astrocytes are sequentially activated within minutes to hours, and leukocytes from the bloodstream, along with their cytokines, accumulate at the lesion site, and even adjacent brain areas, via the damaged blood-brain barrier. These cells further release large amounts of pro-inflammatory factors, exacerbating microglial and peripheral immune cells activation, leading to brain edema and neuronal death. The dead neurons release damage-associated molecular patterns, further intensifying the inflammatory response in the infarct area (Qiu et al., 2021; Yao et al., 2023; Planas, 2024). Therefore, inflammation reduction may be an effective way to prevent PSD development (Lambertsen et al., 2012; Jayaraj et al., 2019; Nagy et al., 2020; Wu et al., 2020; Planas, 2024).

Cilostazol is a phosphodiesterase 3 (PDE3) inhibitor that blocks cAMP degradation, leading to anti-platelet aggregation and vasodilation. It is commonly used to treat conditions such as thrombosis, IS, and thromboangiitis obliterans. Rodent studies have suggested that cilostazol has significant anti-inflammatory effects and potential benefits for brain injury repair. For example, cilostazol can inhibit nuclear factor-κB (NF-κB) activation and tumor necrosis factor-alpha (TNF-α) expression, interleukin (IL)-6), IL-1β, vascular cell adhesion molecule-1, intercellular adhesion molecule-1, and monocyte chemoattractant protein-1 expression (Wang et al., 2008; Sugimoto et al., 2018; de Oliveira Lopes et al., 2022; Saeed et al., 2022). Cilostazol has been reported to increase brain-derived neurotrophic factor secretion (Kim et al., 2016), inhibit indoleamine 2,3-dioxygenase one expression in the hippocampus of mice subjected to underwater trauma (Sadeghi et al., 2024), prevent hippocampus-dependent memory decline and cognitive deterioration in aged mice (Yanai et al., 2018), and reduce neuronal apoptosis by inhibiting the inflammatory response in ischemic regions, thereby promoting neuronal survival and enhancing dopamine neurotransmission (Zaki et al., 2022). Small-scale clinical trials have confirmed that cilostazol can alleviate depressive symptoms in patients with PSD (Nishimura et al., 2007). Moreover, cilostazol has demonstrated significant antidepressant effects in older patients (≥65 years) with major depressive disorder (MDD) insufficiently responding to antidepressant (Baba et al., 2007). A clinical trial demonstrated that cilostazol effectively reduced depression scores in patients with MDD compared with those in the placebo group (Khadivi et al., 2022). These findings indicate cilostazol’s potential as a PSD prevention drug, however its specific mechanisms require further elucidation.

Investigative studies on the pharmacological effects of cilostazol revealed that by inhibiting or blocking cAMP degradation, cilostazol inhibits RhoA activation (Park et al., 2013; Sheu et al., 2014). RhoA is a cAMP-regulated downstream molecule, and increased intracellular cAMP can inhibit RhoA activation (Laudanna et al., 1997; Zieba et al., 2011; Fan et al., 2020; Li L. et al., 2021). Furthermore, RhoA is a small GTPase that acts as a molecular switch in intracellular signal transduction, regulating cell morphology, migration, proliferation, and survival (Tolias et al., 2011; de Seze et al., 2023). RhoA is inactive and active when bound to GDP and GTP, respectively (Vale-Silva et al., 2025). The active form, RhoA-GTP, can bind to inhibitor of κB kinase-γ (IκB-γ), which subsequently activates IκB-β. The IκB complex is a key regulator of NF-κB and comprises the catalytic subunits, IκB-αand IκB-β, and the regulatory subunit, IκB-γ. In a resting state, IκB-α binds to the p65 subunit of NF-κB in the cytoplasm. The C-terminal of IκB contains 3–8 ankyrin repeat sequences that bind to NF-κB, masking the nuclear localization sequence of its Rel homology domain and inhibiting NF-κB activity. Activated IκB-β phosphorylates IκB-α, leading to IκB degradation and p65 translocation (from the cytoplasm to the nucleus), where p65 bind to inflammation-related genes, initiating inflammatory cytokines transcription and triggering inflammation (Kim et al., 2018; Dogsom et al., 2023; Guo et al., 2024; He et al., 2025). RhoA inactivation can inhibit lipopolysaccharide (LPS)-induced NF-κB activation, which clearly indicates that RhoA is a positive upstream regulator of NF-κB (He et al., 2011). Therefore, we hypothesize that cilostazol may reduce inflammation in patients with PSD by inhibiting the RhoA/NF-κB signaling pathway, thereby helping prevent PSD. Therefore, in this study, we conducted a series of experiments using a PSD mouse model and BV2 microglia to explore this hypothesis, focusing on behavioral scores, hippocampal neuronal damage, inflammatory cytokines secretion, and signaling molecule expression.

## 2 Materials and methods

### 2.1 Experimental animals and ethics statement

Eight-week-old male C57BL/6J mice weighing 21 ± 1 g were purchased from Beijing Charles River (China), and housed in pathogen-free temperature-controlled conditions 24 °C ± 1 °C. The mice were divided into the control (Con), PSD, and cilostazol (Cil) groups. All animal procedures were approved by the Animal Ethics Committee of Henan Normal University (Approval No, 2023BS-1026), and all efforts were undertaken to minimize the number of animals.

### 2.2 IS mouse model protocol and cilostazol intervention

The IS mouse model was established using the middle cerebral artery occlusion (MCAO) (Morris et al., 2016; Zhu et al., 2023). The mice were anesthetized using isoflurane and fixed. The neck was disinfected with 75% alcohol, and a longitudinal incision was made to left of the midline using surgical scissors, cutting through the skin and muscle until the arterial pulsation was visible. Blunt dissection of the muscle, and left common, external, and internal carotid arteries was performed using a vascular forceps. Next, the internal carotid artery was clamped, and the proximal end of the common and the external carotid artery were ligated. Subsequently, the common carotid artery bifurcation was incised, a filament was quickly inserted into the internal carotid artery and the distal end of the common carotid artery was ligated (neither tightly nor loosely). The arterial clamp was removed, and the filament was further advanced into the internal carotid artery until a slight resistance was sensed (approximately 10 mm). Finally, the distal end of the common carotid artery was securely ligated, the skin sutured, and the wound disinfected with iodine. After 1 h, the filament was withdrawn, the ligature untied to restore cerebral flow, and the surgical wound sutured. After the mice awakened, the Longa score was assessed, and mice with scores 1–3 were considered IS models (Sun et al., 2020; Wang et al., 2023). The Con group mice underwent only a MCAO sham operation. One week after MCAO, IS mice were subjected to chronic unpredictable mild stress (CUMS) to establish the PSD model (Chen et al., 2013; Kim et al., 2016). The CUMS protocol included the following: horizontal shaking (5 min), cold (4 °C) swimming (3 min), body restraint (2 h), stay in cages tilted at a 45° angle (6 h), continuous illumination at night (12 h), water deprivation (24 h), and wet bedding (24 h). Each of these stressors was applied at different times once a day over a 4-week period (Wang et al., 2023). The CUMS procedure was performed by an independent experimenter who was blinded to the experimental groupings.

**FIGURE 1:**
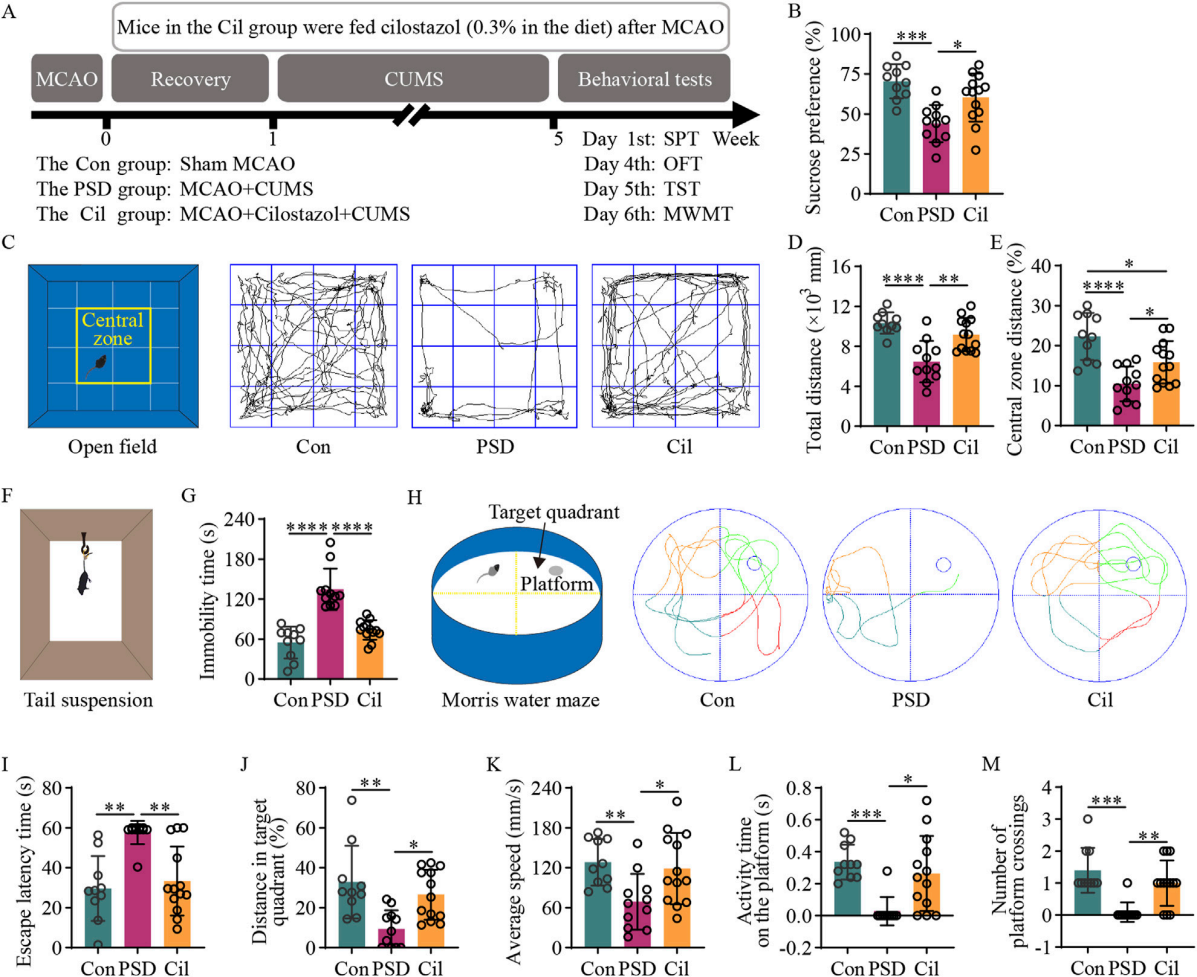
Cilostazol has a significant preventive efficacy against post-stroke depression (PSD) in ischemic stroke (IS) mice under chronic unpredictable mild stress (CUMS). **(A)** A schematic diagram illustrating mouse grouping, modeling, drug intervention, and behavioral testing schedule. The experimental mice are divided into the control (Con), PSD and the cilostazol (Cil) groups. The Con group mice underwent a sham middle cerebral artery occlusion (MCAO). The PSD group mice underwent MCAO to establish the IS model, and those with successful IS modeling were subjected to 4 weeks of CUMS after a 1-week recovery period. The Cil group mice were modeled similarly as those in the PSD group, albeit were treated with cilostazol (0.3% in the diet) post-MCAO. Sucrose preference (SPT), open field (OFT), tail suspension (TST) and Morris water maze (MWMT) tests were conducted on the different groups in the sixth week (Con, n = 10; PSD, n = 11; and Cil, n = 13). **(B)** Sucrose preference in the different groups. **(C)** Schematic diagram of the open field and its central zone, along with representative activity trajectories of the different groups. **(D,E)** Total distance traveled in the open field **(D)** and the percentage of distance traveled in the central zone **(E)** by the different groups. **(F)** Schematic diagram illustrating tail suspension test. **(G)** Immobility time of tail suspension in the different groups. **(H)** Schematic diagram of the escape platform and target quadrant in the Morris water maze, along with representative escape trajectories of the different groups. **(I–M)** Escape latency time **(I)**, percentage of distance traveled in the target quadrant **(J)**, average escape speed **(K)**, activity time on the platform **(L)** and number of platform crossing **(M)** in the different groups of mice. *p < 0.05, **p < 0.01, ***p < 0.001, ****p < 0.0001.

The PSD mice were divided into PSD and Cil group. As previously described in the literature (Oyama et al., 2011; Kasahara et al., 2012), the Cil group mice were administrated cilostazol (H10960014, Zhejiang Otsuka Pharmaceutical, China) from immediately post-MCAO to until tissue collection at the 6-week endpoint (based on a comparison of the dosage mentioned in the literature, 0.3% cilostazol was added to the feed (Oyama et al., 2011; Kasahara et al., 2012; Saito et al., 2014; Yanai et al., 2017)) (Figure 1A).

### 2.3 Behavioral tests

The PSD preventive efficacy of cilostazol in IS mice was evaluated using the sucrose preference (SPT), open field (OFT), tail suspension (TST), and Morris water maze (MWMT) tests (Figure 1A). All behavioral measurements were performed by an observer blinded to experimental groupings.

The SPT was used to evaluate the change of interest. Pre-test, each mouse housed individually. First, the mice underwent a 48-h training for sucrose water adaptation. Two bottles of 1% sucrose water were placed in each cage. After 24 h, one of the bottles was replaced with potable water. After another 24 h, the mice were deprived of water for 12 h, subsequently provided one weighed bottle of potable water and another of 1% sucrose water. After 4 h, both bottles were swapped, and another 4 h, the residual water in both bottles was weighed. The difference in weight was recorded as the 8-h water consumption. Sucrose preference rate = sucrose water consumption/(sucrose water consumption + potable water consumption) × 100%.

The OFT was used to evaluate spontaneous exploratory behavior. First, the mice were placed in the behavioral testing room overnight for adaptation. Each mouse was placed in the open field (JLBehv-LAG1, Shanghai Yuyan Scientific Instruments, China) for 1 min to acclimate. Subsequently, the total and central zone distance traveled in the open field for 5 min were recorded.

The TST was used to evaluate despair levels. First, the mice were placed in the behavioral testing room overnight for adaptation. Subsequently, a bandage was used to secure the tail 1 cm from the tip. The mouse was suspended on the TST device (JLBehv-LG1, Shanghai Yuyan Scientific Instruments) for 6 min, and the immobility time during the last 4 min was recorded.

The MWMT was used to evaluate the learning and memory ability of spatial location. Pre-test, each mouse underwent a 5-day spatial navigation training. First, the mice were placed in the behavioral testing room overnight for adaptation. The Morris water maze (JLBehv-MWMM, Shanghai Yuyan Scientific Instruments) was filled with water 1 cm above the escape platform. The water was dyed white with non-toxic, odorless dye to conceal the platform. The water temperature was maintained at 24 °C. Next, the curtains around the perimeter of the maze were closed, and each mouse placed individually into the maze from the same position. If a mouse did not find the platform within 60 s, it was guided to the platform and held there for 10 s. Each mouse was trained 4 times/day, with a time interval of no less than 30 min, for 5 days. On the sixth day, the platform was removed, and the time required the mice to reach the platform location within 60 s and the time and distance of activities in the quadrant area of the platform location were recorded. The spatial memory ability of the mice was evaluated by comparing the latency time (from the time when mice entered the water to the time when they first crossed the platform), the percentage of distance in the target quadrant, average speed, activity time on the platform and number of platform crossings.

The OFT, TST, and MWMT parameters were recorded using the Digbehv 4.1.8 video analysis system.

### 2.4 Cryosectioning, nissl staining and immunofluorescence labeling of mouse brain tissue

Post-completion the behavioral tests, some of the mice were perfused with saline. When the saline from the right atrium was clear and blood-free, the mice were further perfused with 4% paraformaldehyde. Once the mice exhibited signs of rigor mortis, the brain tissue was dissected and fixed in 4% paraformaldehyde, dehydrated with gradient sucrose solution (15%–20%–30%), embedded in OCT compound (4,583, SAKURA, USA), and rapidly frozen in liquid nitrogen. Subsequently, the brain tissues were sectioned at 10 μm/slice using a cryostat (CM 1950, Leica, Germany) and placed on a flattening table (HI1220, Leica) at 37 °C overnight.

Some sections were washed twice with distilled water (2 min each), Nissl stained (C0117, Beyotime Biotechnology, China) for 10 min, and again washed twice with distilled water (5 s each). Next, they were immersed in 95% ethanol thrice (5 s, 2 min, 2 min, respectively), cleared with xylene twice (5 min each), and mounted with neutral resin.

Other sections, or some cultured cells, were rinsed thrice with phosphate-buffered saline (PBS) (5 min each), permeabilized with 0.3% Triton X-100 (HFH10, Thermo Fisher Scientific, USA) for 30 min, and again rinsed thrice with PBS (5 min each). The samples were blocked with goat serum (C0265, Beyotime Biotechnology) at 25 °C for 1 h. After removing the serum without washing, primary antibodies were added (Anti-NeuN antibody, 24,307, Cell Signaling Technology, USA; Anti-Caspase-3 antibody, sc-7272, Santa Cruz Biotechnology, USA; Anti-IBA1 antibody, 17,198, Cell Signaling Technology; Anti-MAP2 antibody, 4542S, Cell Signaling Technology) and incubated overnight at 4 °C. The samples were subsequently rinsed thrice with PBS (5 min each), and fluorophore-labeled secondary antibodies were added (Goat Anti-Rabbit IgG H&L/FITC, ab6717, Abcam, UK; Goat Anti-Mouse IgG H&L/Alexa Fluor^®^ 647, ab150115, Abcam) and incubated in a humidified chamber at 37 °C for 1 h. After rinsing thrice with PBS (5 min each), the samples were counterstained with DAPI (C1006, Beyotime Biotechnology) for 10 min, rinsed thrice with PBS (5 min each), and mounted with an antifade mounting medium (P0128, Beyotime Biotechnology), observed and photographed using an upright or an inverted fluorescence microscope (Eclipse Ni-U, Nikon, Japan or DMi 8, Leica, respectively), and analyzed using the ImageJ software.

### 2.5 Culturing and transfection of mouse microglial BV2 microglia

Mouse BV2 microglia (10,810-BV2, CytoBiotech, China) were cultured at 37 °C in a 5% CO_2_ incubator using high-glucose Dulbecco’s Modified Eagle Medium (DMEM; 11,965,092, Gibco, USA) supplemented with 10% fetal bovine serum (FBS; 10100147, Gibco) and 1% penicillin-streptomycin solution (C0222, Beyotime Biotechnology). The cells were maintained until they reached the logarithmic growth phase with a confluence of 80%–90%, after which they were either passaged or used for subsequent experiments.

Well-grown BV2 microglia were counted and evenly seeded into 6-well culture plates. Once the cells reached approximately 80% confluence, the medium was replaced with fresh antibiotic-free complete medium. Following the manufacturer’s instructions for Lipofectamine 3,000 (L3000001, Thermo Fisher Scientific), 3 μg of pcDNA-RhoA expression plasmid (prepared by GeneChem, China) was transfected into the BV2 microglia. After 48 h, the cells were subjected to G418 selection for 24 h to obtain BV2 microglia with stable RhoA overexpression.

### 2.6 Determination of cilostazol effects on RhoA-overexpressed BV2 microglia

To determine the appropriate cilostazol treatment concentration, RhoA-overexpressed BV2 microglia were seeded in a 96-well plate and treated with 0, 5, 10, 20, 40 or 80 μM cilostazol (S1294, Selleck, USA) dissolved in dimethyl sulfoxide (DMSO; D2650, Sigma-Aldrich, USA) for 24 h. Next, 10 μL CCK-8 reagent (C0038, Beyotime Biotechnology) was added to each well, and the plate was incubated at 37 °C for 30 min. Absorbance was measured at 450 nm using a microplate reader (Varioskan *LUX*, Thermo Fisher Scientific) to evaluate cell viability.

Based on previously described methods (Altun-Gultekin et al., 1998; Wei et al., 1998; Ren et al., 1999; Toksoz and Merdek, 2002) and the Rho Activation Assay Biochem Kit™ (BK036, Cytoskeleton, USA) protocol, FBS addition or the use of fibronectin-coated plates led to RhoA activation in the cultured cells. Specifically, 10% and 5% FBS increased RhoA activation to two to six and 2 times the basal (serum free) level, respectively. Since lower FBS concentrations may impair long-term cell survival, cells cultured with 1%–5% and 10% FBS can be used as the control and RhoA activation group, respectively. When the cells were seeded onto fibronectin-coated plates (1% FBS for culture), RhoA, not activated during the first 10–20 min, gradually became activated to 1–6 times the basal level. Peak RhoA activation occurred between 60 and 90 min, returning to basal levels within 6 h. In this study, various concentrations of FBS and fibronectin-coated plates (J00645, Jingan Biological, China) were used to culture BV2 microglia and modulate RhoA activation with cilostazol intervention. The experimental grouping and processing methods were as follows:

Con1, normal BV2 cells cultured in a medium containing 5% FBS; Vehicle, BV2 cells transfected with empty plasmid cultured in the 5% FBS medium; Con1+RhoA, RhoA-overexpressed BV2 cells cultured in the 5% FBS medium; RhoA1, RhoA-overexpressed BV2 cells inoculated into fibronectin-coated plates and cultured in the 10% FBS medium; RhoA1+Sol, RhoA1, and DMSO-treated; RhoA1+Cil, RhoA1, and cilostazol-treated.

The DMSO dose in the RhoA1+Sol and RhoA1+Cil groups were identical. In the RhoA1+Cil group, 10 μM cilostazol (S1294, Selleck; DMSO dissolved) was added to the culture medium. After 12 h, all cells and culture supernatants were collected. The effects of cilostazol on the total RhoA, activated RhoA (RhoA-GTP), and NF-κB p65 expression were analyzed using Western blot, quantitative polymerase chain reaction (qPCR), and RhoA pull-down assays. The impact of cilostazol on TNF-α and IL-1β secretion by BV2 microglia was measured via an enzyme-linked immunosorbent assay (ELISA).

### 2.7 High-performance liquid chromatography (HPLC)

Cells from the aforementioned groups were harvested. Ice-cold (4° C) 0.15 M perchloric acid (10 μL/mg of cells) was added to the cell pellets and homogenized for 20 s using an ultrasonic cell disruptor (JY92-2D, Ningbo Scientz Biotechnology, China). The homogenates were centrifuged at 15,000 rpm for 20 min at 4 °C. The resulting supernatant was filtered through a 0.22 μm membrane for subsequent HPLC analysis.

The cAMP standard (A9501, Sigma-Aldrich) was dissolved in a 50% methanol-water solution. Standard curves were constructed, and the samples were analyzed using an LC-20A DXR ultra-fast HPLC system (Shimadzu, Japan) to determine cAMP levels based on peak areas. Regarding the chromatographic conditions, a C18 column (Inertsil ODS-SP 4.6 × 150 mm, 5 μm, Shimadzu) was used with a mobile phase comprising 18% methanol and 82% KH_2_PO_4_ (0.02 M), the flow rate was maintained at 0.8 mL/min with an injection volume of 20 μL, the column temperature maintained at 25 °C, and detection was performed at 260 nm.

### 2.8 Primary hippocampal neurons culture and cilostazol intervention

Based on previously described methods (Volpicelli-Daley et al., 2011; Ma et al., 2023), hippocampi from neonatal mice (0–1-day-old) were isolated and placed in trypsin (R001100, Gibco) at 37 °C for 15 min. Next, the digestion was ceased using high-glucose DMEM (11965092, Gibco) containing 10% FBS (10100147, Gibco), and the tissue was filtered through a cell strainer (258,367, NEST, China). After centrifugation at 1,000 rpm for 5 min, the supernatant was discarded, and the cells were re-suspended in the culture medium. Primary hippocampal neurons were seeded at the desired density onto poly-d-lysine-coated (A3890401, Gibco) six- or 24-well plates, based on the requirements of different experiments. The cells were cultured in Neurobasal™-A medium (10888022, Gibco) supplemented with 2% B27 (A1895601, Gibco) and 1× GlutaMAX™ (35050061, Gibco) (complete primary neuron culture medium; hereinafter, culture medium). After 4–6 h, the medium was replaced with fresh culture medium, and the cells were incubated in a humidified incubator at 37 °C with 5% CO_2_. The medium was half-replaced with fresh culture medium on the rthird day. These primary hippocampal neurons were used for subsequent experiments on the fifth day.

Normal and RhoA-overexpressed BV2 cells were inoculated into a 6-well plate. The treatment methods of each group of cells are as mentioned in Section 2.6. The culture medium was collected for the culture of primary hippocampal neurons. Neuron cell grouping and processing methods were as follows:

Con2, primary neurons cultured in the culture medium; Con2+RhoA, primary neurons cultured in 50% culture medium +50% culture supernatant from the Con1+RhoA group.

RhoA2, primary neurons cultured in 50% culture medium +50% culture supernatant from the RhoA1 group; RhoA2+Cil, primary neurons cultured in 50% culture medium +50% culture supernatant from the RhoA1+Cil group; RhoA2++Cil, primary neurons cultured in 50% culture medium +50% culture supernatant from the RhoA1 group +10 μM cilostazol.

The culture medium in each group was half-replaced every 24 h. After 7 days of continuous culture, neuronal morphology and apoptosis were observed. Using the concentric circle (Sholl’s) analysis in the ImageJ software, neuronal branching complexity was indirectly evaluated by counting the number of intersections between the neuronal branches and concentric circles per radius (increased by 100 pixels, approximately 6 μm).

### 2.9 Western blot

hippocampus samples, from mice after the behavioral tests or cultured cells, were harvested and homogenized (DS1000, New Zong Ke Viral Disease Control Bio-Tech, China) in a RIPA lysis buffer (P0013B, Beyotime Biotechnology) containing 1 mM PMSF protease inhibitor. Next, the samples were lysed on ice for 30 min, and centrifuged at 12,000 rpm for 10 min at 4 °C. The supernatant was collected, and the total protein concentration was measured using a BCA protein assay kit (P0010S, Beyotime Biotechnology). Subsequently, an SDS-PAGE loading buffer was added to the samples proportionally, and the samples were boiled for 5 min using dry bath incubator (OSE-DB-02, Tiangen Biotech, China).

The proteins were separated via electrophoresis (PowerPac Basic, BIO-RAD, USA and Mini-PROTEAN^®^ Tetra System, BIO-RAD). The target proteins were transferred from the gel to a PVDF membrane (FFP77, Beyotime Biotechnology) using a semi-dry transfer system (Trans-Blot^®^ Turbo™ BIO-RAD). The PVDF membrane was washed thrice with PBST (5 min each), blocked with BSA (A8020, Solarbio, China) at 25°C for 2 h, and subsequently incubated with primary antibodies (Anti-β-actin antibody, ab9485, Abcam; Anti-Bcl-2 antibody, 68103-1-Ig, Proteintech, China; Anti-Bax antibody, 50599-2-Ig, Proteintech; Anti-TNF-α antibody, ab183218, Abcam; Anti-IL-1β antibody, ab254360, Abcam; Anti-RhoA antibody, ab187027, Abcam; Anti-NF-κB p65 antibody, ab32536, Abcam) overnight at 4 °C. The next day, the membrane was washed thrice with PBST (10 min each) and incubated with HRP-conjugated secondary antibodies (Goat anti-rabbit IgG H&L/HRP, ab6721, Abcam) at 25 °C for 1 h. The membrane was washed thrice with PBST (5 min each), and ECL substrate (638,173, Millipore, USA) was added for detection. Protein expression was visualized using an ultra-sensitive multifunctional imager (Amersham Imager 600, GE life sciences, USA) and analyzed using the ImageJ software.

### 2.10 RhoA pull-down assay

Based on previously described methods (Lu et al., 2022), the hippocampus samples or cultured cells were lysed, and RhoA-GTP levels were determined using the Rho Activation Assay Biochem Kit™ according to the manufacturer’s protocol.

### 2.11 qPCR

Hippocampus samples or cultured cells were harvested and homogenized in 50–100 mg/mL Trizol (T9424, Sigma-Aldrich). Next, the samples were placed on ice for 5 min and centrifuged at 12,000 rpm for 5 min at 4 °C. The supernatant was collected, and 200 μL of chloroform was added. The mixture was thoroughly shaken, placed on ice for 5 min, and centrifuged at 12,000 rpm for 15 min at 4 °C. The aqueous phase was collected, mixed gently with an equal volume of isopropanol, and placed at 25 °C for 10 min. After centrifuging at 12,000 rpm for 10 min at 4 °C, the supernatant was discarded, and the pellet was washed with 75% ethanol and dried completely. Subsequently, the RNA was dissolved in an appropriate amount of diethylpyrocarbonate water, and the RNA concentration was measured using a NanoDrop 2000 spectrophotometer (Thermo Fisher Scientific).

The RNA was reverse-transcribed into cDNA using the NovoScript^®^ Plus All-in-one first Strand cDNA Synthesis SuperMix (gDNA Purge) (E047, Novoprotein, China) kit. The synthesized cDNA was amplified using PowerUp SYBR Green Master Mix (A25741, Thermo Fisher Scientific) in a total reaction volume of 20 μL (10 μL of 2× PowerUp SYBR Green Master Mix, 1 μL of forward primer, 1 μL of reverse primer, 2 μL of cDNA, and 6 μL of water). The reaction conditions were as follows: Uracil DNA Glycosylase (UDG) activation at 50 °C for 2 min, initial denaturation at 95 °C for 2 min, followed by 40 cycles of denaturation at 95 °C for 15 s and annealing/extension at 60 °C for 1 min. The relative mRNA levels were calculated using the 2^−ΔΔCT^ method. The primers were synthesized by Sangon Biotech (China), and the sequences are as follows:

β-actin-F, 5′- ACT​GCC​GCA​TCC​TCT​TCC​T-3’; β-actin-R, 5′- TCA​ACG​TCA​CAC​TTC​ATG​ATG​GA-3’; RhoA-F, 5′-AGC​TTG​TGG​TAA​GAC​ATG​CTT​G-3’; RhoA-R, 5′-GTG​TCC​CAT​AAA​GCC​AAC​TCT​AC-3’; TNF-α-F, 5′-CCT​GTA​GCC​CAC​GTC​GTA​G-3’; TNF-α-R, 5′-GGG​AGT​AGA​CAA​GGT​ACA​ACC​C-3’; IL-1β-F, 5′-GCC​CAT​CCT​CTG​TGA​CTC​AT-3’; IL-1β-R, 5′-AGG​CCA​CAG​GTA​TTT​TGT​CG-3’; Bcl-2-F, 5′-TTC​GGT​GTA​ACT​AAA​GAC​AC-3’; Bcl-2-R, 5′-CTC​AAA​GAA​GGC​CAC​AAT​CC-3’; Bax-F, 5′-GGA​ATT​CGC​CGT​GAT​GGA​CGG​GTC​CGG-3’; Bax-R, 5′-GGA​ATT​CTC​AGC​CCA​TCT​TCT​TCC​AGA-3’.

### 2.12 ELISA

The cell culture supernatant was harvested and centrifuged at 3,000 rpm for 5 min at 4 °C to remove cell debris. TNF-α and IL-1β levels in the supernatant of the RhoA-expressing or -activated BV2 microglia, as well as in the various treatment groups from subsequent experiments, were measured using TNF-α (SCA133Mu, Cloud-clone, China) and IL-1β (SEA073Mu, Cloud-clone) ELISA kits.

### 2.13 Statistical analysis

All data were presented as mean ± standard deviation (SD). The GraphPad Prism 9.0 software (GraphPad Software, USA) was used for statistical analysis and mapping. For between-group comparisons, two-tailed unpaired *t*-tests were used for normal distributions, while the Mann–Whitney *U*-test was applied for non-normal distributions (although no encountered in the present study). For analyses involving ≥3 groups, data sets were tested for normality, homogeneity of variance, and sphericity assumptions before performing one- or two-way analysis of variance (ANOVA). Normality was assessed using the Shapiro–Wilk test (p > 0.05 indicating normal distribution). Normal data sets underwent homogeneity of variance analysis, while non-normal data sets were analyzed using the Kruskal–Wallis test followed by Dunn’s multiple comparisons tests. Homogeneity of variance was examined using the Brown–Forsythe test (p > 0.05 indicating equal variances). Data sets not meeting this assumption were analyzed using Welch ANOVA tests followed by Dunnett’s T3 multiple comparisons tests. For repeated measures analyses, Mauchly’s test of sphericity was additionally performed (p > 0.05 indicating satisfaction of sphericity assumption, else Greenhouse-Geisser correction was applied). For data sets satisfying both normality and homogeneity of variance assumptions, one- or two-way ANOVA was performed followed by Tukey’s *post hoc* multiple comparisons test to determine statistical significance. All statistical tests were two-tailed, with p < 0.05 considered statistically significant.

## 3 Results

### 3.1 Cilostazol has a significant preventive efficacy against PSD

The SPT results (Figure 1B) revealed that sucrose preference in the PSD group was significantly lower than that in the Con group (F_2, 31_ = 11.570, p = 0.0001). However, the Cil group exhibited a significant higher sucrose preference than the PSD group (F_2, 31_ = 11.570, p = 0.0106). No significant between-group difference was observed (F_2, 31_ = 11.570, p = 0.1596). These results indicate that cilostazol can significantly increase the sucrose water consumption in CUMS-subjected IS mice, enhancing their sensitivity and excitability to sweetness.

The OFT results (Figures 1C–E) revealed that compared with the Con group, the PSD group had a significantly decreased total distance (F_2, 31_ = 15.140, p < 0.0001) and percentage of central zone distance (F_2, 31_ = 13.640, p < 0.0001). Conversely, the Cil group exhibited a significantly increase total distance (F_2, 31_ = 15.140, p = 0.0012) and percentage of central zone distance (F_2, 31_ = 13.640, p = 0.0440) compared with the PSD group. No significant difference was observed in total distance between the Cil and Con groups (F_2, 31_ = 15.140, p = 0.2339); however, the percentage of central zone distance remained significantly decreased in the Cil group (F_2, 31_ = 13.640, p = 0.0154). These results suggest that cilostazol helps enhance spontaneous activity and exploratory behavior in CUMS-subjected IS mice.

The TST results (Figures 1F,G) indicated that the PSD group had a significantly higher immobility time than the Con group (F_2, 31_ = 33.880, p < 0.0001). However, the Cil group had a significantly lower immobility time than the PSD group (F_2, 31_ = 33.880, p < 0.0001), with no significant difference compared with the Con group (F_2, 31_ = 33.880, p = 0.1587). These findings suggest that cilostazol markedly increases tail suspension struggling behavior in CUMS-subjected IS mice, significantly alleviating their sense of despair.

The MWMT results (Figures 1H–M) revealed that compared with the Con group, the PSD group had a significantly increased escape latency time (H = 14.670, p = 0.0012) and significantly decreased percentage of distance in the target quadrant (F_2, 31_ = 8.570, p = 0.0012), average speed (F_2, 31_ = 5.474, p = 0.0136), activity time on the platform (H = 14.510, p = 0.0007) and number of platform crossings (H = 17.500, p = 0.0002). Conversely, the Cil group exhibited a significantly decreased escape latency time (H = 14.670, p = 0.0069) and significantly increased percentage of distance in the target quadrant (F_2, 31_ = 8.570, p = 0.0117), average speed (F_2, 31_ = 5.474, p = 0.0291), activity time on the platform (H = 14.510, p = 0.0159) and number of platform crossings (H = 17.500, p = 0.0053) compared with the PSD group. No significant differences were observed in escape latency time (H = 14.670, p > 0.999), percentage of distance in the target quadrant (F_2, 31_ = 8.570, p = 0.5197), average speed (F_2, 31_ = 5.474, p = 0.8700), activity time on the platform (H = 14.510, p = 0.7840) and number of platform crossings (H = 17.500, p = 0.8273) between the Cil and Con groups. These results indicate that cilostazol can significantly enhance escape desire in CUMS-subjected IS mice and improve their cognitive accuracy in locating the escape platform.

Overall, the results confirm that cilostazol can alleviate depression-like behavior in CUMS-subjected IS mice and has a significant preventive efficacy against PSD.

### 3.2 Cilostazol mitigates hippocampal neuron damage and apoptosis

**FIGURE 2:**
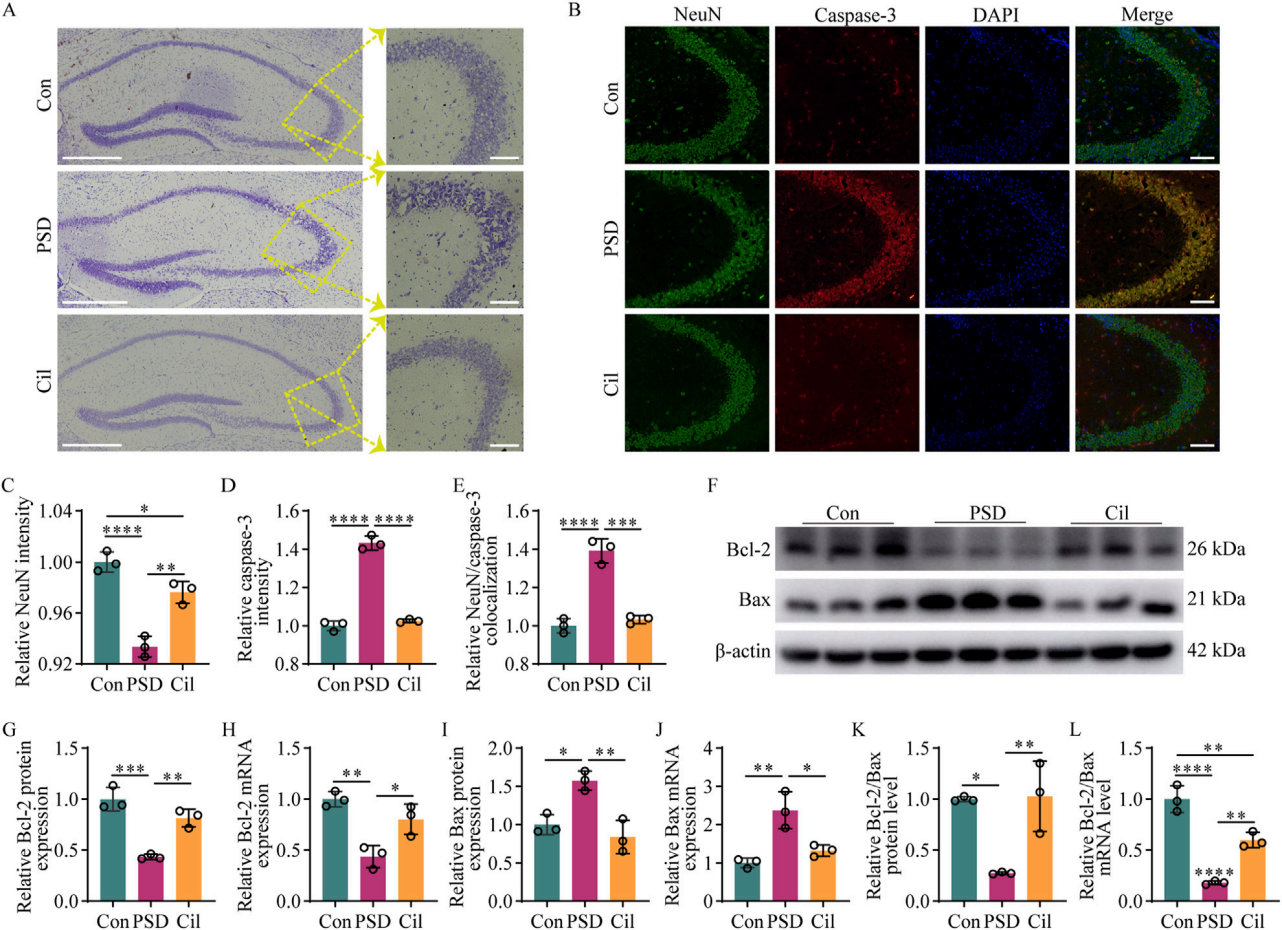
Cilostazol mitigates hippocampal neuron damage and apoptosis in ischemic stroke mice under chronic unpredictable mild stress (n = 3). **(A)** Representative photomicrographs (scale bars: left, 500 μm and right, 100 μm) of Nissl staining in hippocampal tissues from the different groups of mice. In the control (Con) group, the neurons are arranged in a regular and dense pattern, with nearly round cell bodies that are uniformly blue, with lightly stained nuclei. In the post-stroke depression (PSD) group, the neurons are arranged disorderly, with diverse cell morphologies and variable stain intensities. The cilostazol (Cil) group has a similar neuronal arrangement, morphology, and overall cell body staining as that in the Con group, albeit with fewer neurons with lightly stained nuclei. **(B)** Representative photomicrographs (scale bars: 100 μm) of immunofluorescence labeling in hippocampal tissues from the different groups. NeuN, as a marker for normal neurons, appearing green. Caspase-3, as a marker for apoptotic cells, appearing red. DAPI, as a fluorescent dye that binds to cell nuclei, appearing blue. The “Merge” image combines three kinds of fluorescence into a composite image, depicting the colocalization of NeuN, caspase-3, and neurons. **(C–E)** Fluorescence intensity analysis of NeuN **(C)**, caspase-3 **(D)**, and their colocalization **(E)**. NeuN and caspase-3 expression levels are represented by fluorescence intensity, and the colocalization of NeuN and caspase-3 is analyzed based on the overlap of their fluorescence areas. **(F)** Representative blots exhibiting Bcl-2 and Bax protein relative expression levels in the hippocampal tissues from the different groups. **(G–J)** Analysis of Bcl-2 protein **(G)**, Bcl-2 mRNA **(H)**, Bax protein **(I)**, and Bax mRNA **(J)** relative expression levels in the hippocampal tissues from the different groups. **(K–L)** Analysis of Bcl-2/Bax value in the hippocampal tissues from the different groups, separately for protein **(K)** and mRNA **(L)** relative expression levels. *p < 0.05, **p < 0.01, ***p < 0.001, ****p < 0.0001.

Nissl staining results (Figure 2A) revealed that in the Con group, the hippocampal neurons were arranged in a regular and dense pattern, with nearly round cell bodies, with uniformly blue and lightly stained nuclei, indicating that the Nissl body activity was generally at a normal level. In the PSD group, the neurons were disordered, with diverse cell morphologies, and many neurons exhibited significant swelling or rupture, accompanied by irregular large cytoplasmic vacuoles and variable staining intensities, suggesting severe neuronal damage. The Cil group had a similar neuronal arrangement, morphology, and overall cell body staining as that in the Con group, albeit with fewer neurons, with lightly stained nuclei. This could be due to increased heterochromatin (which stain blue) in some neuronal nuclei (which normally have more euchromatin and lighter staining for active gene expression) or a larger distribution of Nissl bodies (which stain blue) in the cell, suggesting that some neurons might remain in a state of stress resistance. These findings indicate that cilostazol helps mitigate the damage to hippocampal neurons in CUMS-subjected IS mice.

Immunofluorescence labeling results (Figures 2B–E) revealed that in the Con group, nearly all neurons exhibited high NeuN and low caspase-3 expressions. Compared with the Con group, the PSD group neurons exhibited significantly decreased NeuN expression (F_2, 6_ = 50.250, p = 0.0002), increased caspase-3 expression (F_2, 6_ = 252.800, p < 0.0001), and increased colocalization of NeuN and caspase-3 (F_2, 6_ = 71.890, p < 0.0001). NeuN expression was significantly higher in Cil group than in the PSD group (F_2, 6_ = 50.250, p = 0.0017) albeit lower than that in the Con group (F_2, 6_ = 50.250, p = 0.0284). Caspase-3 expression was markedly lower in the Cil group than in the PSD group (F_2, 6_ = 252.800, p < 0.0001) and similar to that in the Con group (F_2, 6_ = 252.800, p = 0.5021). Colocalization of NeuN and caspase-3 was significantly lower in the Cil group than in the PSD group (F_2, 6_ = 71.890, p = 0.0002) and not significantly different from that in the Con group (F_2, 6_ = 71.890, p = 0.6618). These findings indicate that cilostazol can significantly reduce hippocampal neuronal apoptosis of in CUMS-subjected IS mice.

Furthermore, Western blot and qPCR results (Figures 2F–L) revealed that, Bcl-2 (anti-apoptotic factor) protein (F_2, 6_ = 33.880, p = 0.0005) and Bcl-2 mRNA (F_2, 6_ = 18.680, p = 0.0023) levels were significantly decreased in the PSD group compared with those in the Con group, while Bax (pro-apoptotic factor) protein (F_2, 6_ = 16.840, p = 0.0119) and Bax mRNA (F_2, 6_ = 17.340, p = 0.0032) levels were significantly increased. In the Cil group, Bcl-2 protein and Bcl-2 mRNA levels were significantly higher than those in the PSD group (F_2, 6_ = 33.880, p = 0.0039; F_2, 6_ = 18.680, p = 0.0184), albeit not significantly different from those in the Con group (F_2, 6_ = 33.880, p = 0.0848; F_2, 6_ = 18.680, p = 0.1680). Conversely, Bax protein and Bax mRNA levels were significantly lower in the Cil group than in the PSD group (F_2, 6_ = 16.840, p = 0.0036; F_2, 6_ = 17.340, p = 0.0120) and similar to those in the Con group (F_2, 6_ = 16.840, p = 0.4925; F_2, 6_ = 17.340, p = 0.4338). The Bcl-2/Bax value can more accurately reflect anti-apoptotic capability. The Bcl-2/Bax value was significantly lower in the PSD group than in the Con group at both protein (F_2, 6_ = 13.700, p = 0.0103) and mRNA (F_2, 6_ = 65.540, p < 0.0001) levels. The Bcl-2/Bax value was significantly higher in the Cil group than in the PSD group at both protein (F_2, 6_ = 13.700, p = 0.0086) and mRNA (F_2, 6_ = 65.540, p = 0.0027) levels and similar to that in the Con group at the protein level (F_2, 6_ = 13.700, p = 0.9840), albeit significantly lower at the mRNA level (F_2, 6_ = 65.540, p = 0.0033). These findings indicate that cilostazol can mitigate hippocampal neuronal apoptosis in CUMS-subjected IS mice.

### 3.3 Cilostazol inhibits the activity of hippocampal microglia

**FIGURE 3:**
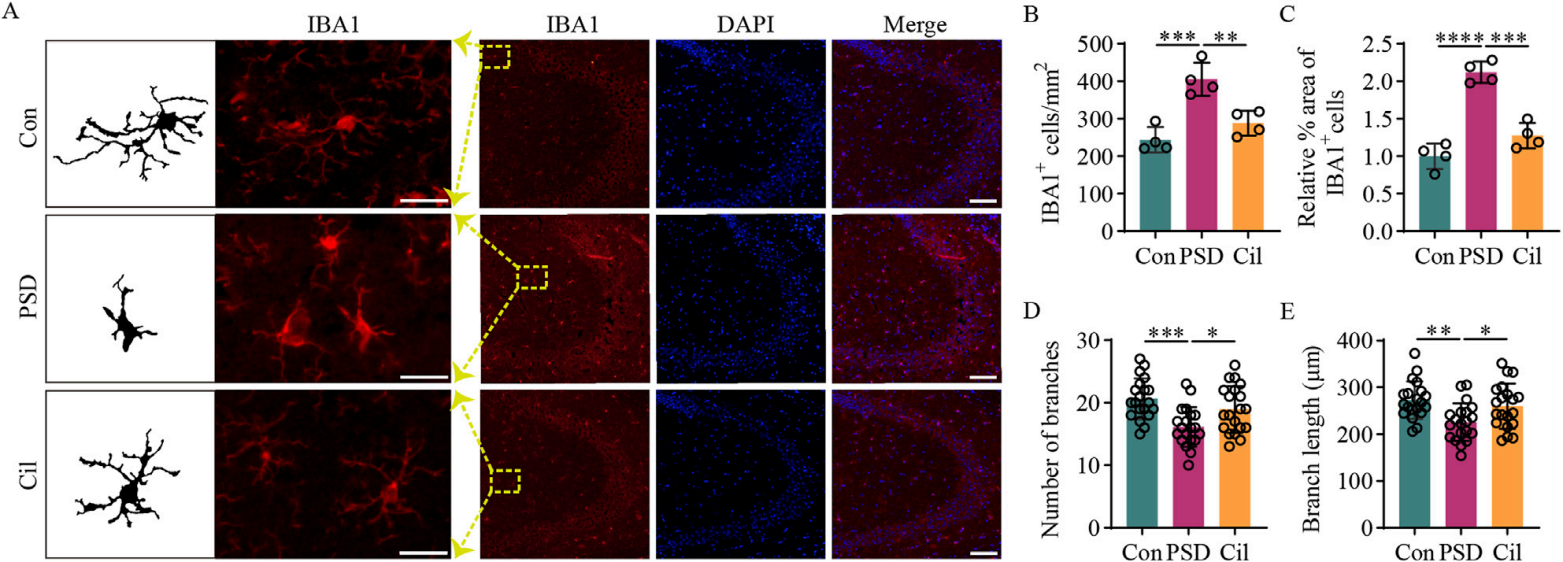
Cilostazol inhibits the activity of hippocampal microglia in ischemic stroke mice under chronic unpredictable mild stress. **(A)** Representative photomicrographs (scale bars: left, 20 μm and right, 100 μm) of immunofluorescence labeling in hippocampal tissues from the different groups of mice (n = 4). IBA1, a marker for microglia, appears red and displays the state and distribution of microglia. DAPI, a fluorescent dye that binds to cell nuclei, appears blue and displays the distribution of all cells. The “Merge” image combines both fluorescence types into a composite image, depicting the proportion of microglia. **(B)** Analysis of microglia counts in the hippocampal tissues from the different groups (n = 4). **(C)** Analysis of the relative IBA1-positive microglia proportion in the hippocampal tissues from different groups (n = 4). **(D)** Analysis of the branch number per microglia in the hippocampal tissues from the different groups (n = 20). **(E)** Analysis of the branch length per microglia in the hippocampal tissues from the different groups (n = 20). *p < 0.05, **p < 0.01, ***p < 0.001, ****p < 0.0001.

Our previous reviews inferred that depression-induced neuronal damage is related to the excessive microglial activation and increased pro-inflammatory cytokines levels (Zhang et al., 2024b; Zhang and Tang, 2024). The results of the investigation on the role inflammation regulation in cilostazol’s PSD prevention mechanism revealed (Figure 3) fewer microglia in the Con group, most of which were in a normal resting state, with tertiary and quaternary branching structures visible on the cell processes. The PSD group had a significantly higher IBA1-positive areas (F_2, 9_ = 52.620, p < 0.0001) and number of microglia (F_2, 9_ = 19.850, p = 0.0005), along with fewer branches (F_2, 57_ = 8.972, p = 0.0003) and shorter branch length (F_2, 57_ = 6.455, p = 0.0028), than the Con group. Most of the microglia in the PSD group were in an activated state, characterized by enlarged, round or rod-shaped cell bodies. The IBA1-positive areas and number of microglia were lower in the Cil group than in the PSD group (F_2, 9_ = 52.620, p = 0.0001; F_2, 9_ = 19.850, p = 0.0043) and similar to those in the Con group (F_2, 9_ = 52.620, p = 0.0885; F_2, 9_ = 19.850, p = 0.2659). The number of branches and branch length were higher than those in the PSD group (F_2, 57_ = 8.972, p = 0.0306; F_2, 57_ = 6.455, p = 0.0383) and comparable with that in the Con group (F_2, 57_ = 8.972, p = 0.2605; F_2, 57_ = 6.455, p = 0.6049), with most microglia in a normal resting state and fewer in an activated state. These results indicate that cilostazol can inhibit the activity of hippocampal microglial activity in CUMS-subjected IS mice, thus mitigating hippocampal neuronal damage and apoptosis.

### 3.4 Cilostazol inhibits RhoA/NF-κB signaling pathway activation and pro-inflammatory cytokines expression

**FIGURE 4:**
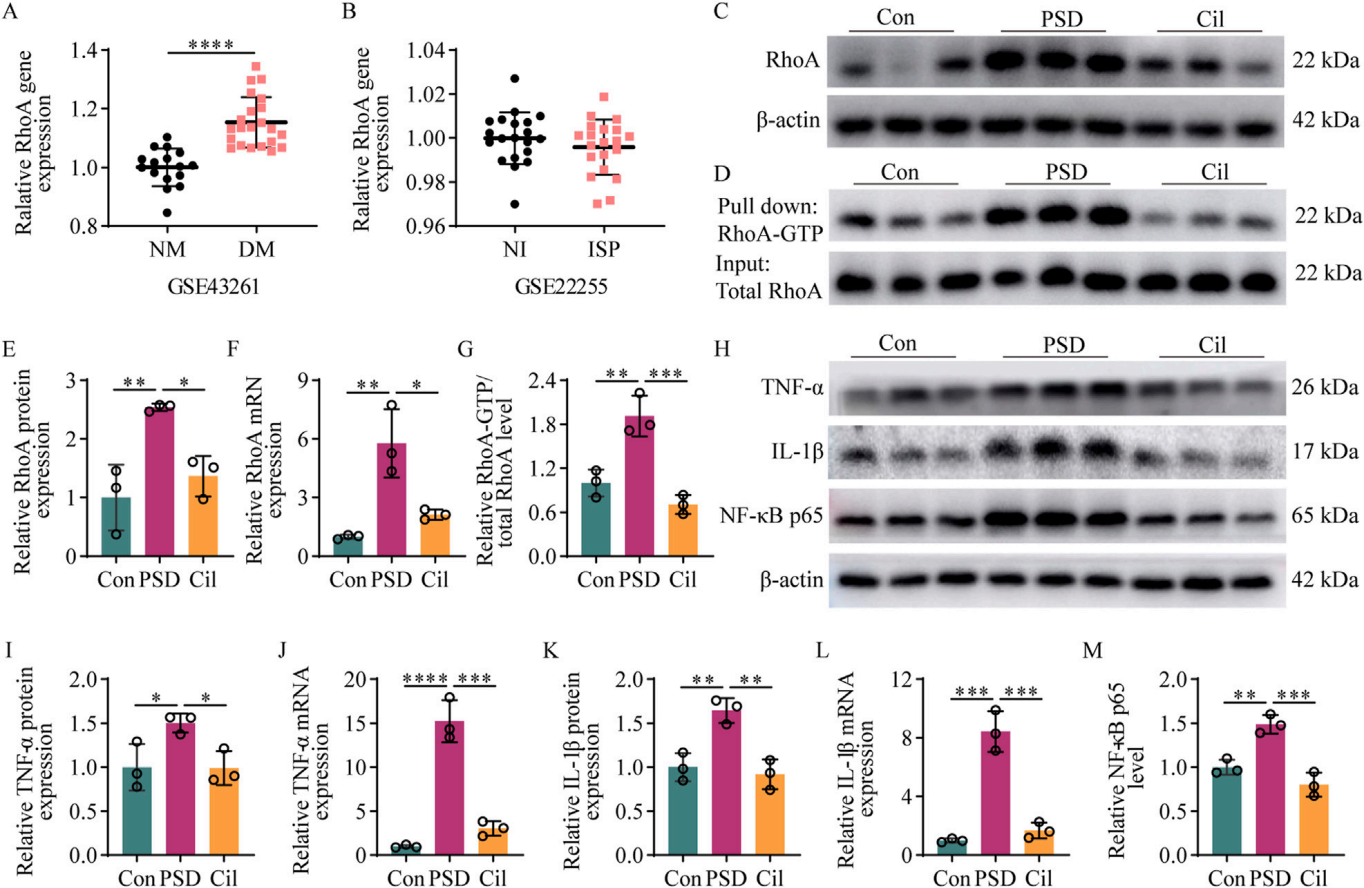
Cilostazol inhibits RhoA/NF-κB signaling pathway activation and pro-inflammatory cytokine expression in ischemic stroke (IS) mice under chronic unpredictable mild stress (n = 3). **(A)** Difference in RhoA gene expression between depressive mice (DM) and normal mice (NM) retrieved from the GSE43261 database. **(B)** Difference in RhoA gene expression between patients with IS (ISP) and normal individuals (NI) retrieved from the GSE22255 database. **(C,D)** Representative blots exhibiting the relative expression **(C)** and activation **(D)** levels of RhoA protein in the hippocampal tissues from the different groups. **(E–G)** Analysis of RhoA protein **(E)** and RhoA mRNA **(F)** relative expression levels, and RhoA protein **(G)** activation levels in the hippocampap tissues from the different groups. **(H)** Representative blots exhibiting TNF-α, IL-1β, and NF-κB p65 protein relative expression levels in the hippocampal tissues from the different groups. **(I–M)** Analysis of TNF-α protein **(I)**, TNF-α mRNA **(J)**, IL-1β protein **(K)**, IL-1β mRNA **(L)**, and NF-κB p65 subunit **(M)** relative expression levels in the hippocampal tissues from the different groups. *p < 0.05, **p < 0.01, ***p < 0.001, ****p < 0.0001.

Regarding the potential mechanisms by which cilostazol inhibits hippocampal microglia activity, cilostazol exerts its effects by inhibiting PDE3 activity, thereby blocking intracellular cAMP degradation. Elevated cAMP levels can inhibit RhoA activation via the PKA signaling pathway (Aslam et al., 2010; Li L. et al., 2021), and RhoA is an upstream activator of NF-κB (He et al., 2011), which is directly related to microglial activation. Additionally, a GEO database search for sequencing depression and stroke-related results revealed that RhoA expression is elevated in depressed mice compared with normal mice (Figure 4A, *t =* 6.005, *df =* 36.000, p < 0.0001) (GSE43261), albeit exhibits no difference between patients with IS and normal individuals (Figure 4B, *t =* 1.085, *df =* 38.000, p = 0.2847) (GSE22255). These findings suggested that RhoA activation in microglia may be closely associated with PSD onset and could be a key molecular target for cilostazol in preventing PSD.

Results of the investigation of RhoA protein, RhoA mRNA, and RhoA-GTP levels in the hippocampus in each mice group, to confirm the aforementioned speculation (Figures 4C–G), revealed that the PSD group had significantly elevated RhoA protein (F_2, 6_ = 13.280, p = 0.0063), RhoA mRNA (F_2, 6_ = 18.000, p = 0.0029), and RhoA-GTP (F_2, 6_ = 28.000, p = 0.0039) levels compared with the Con group. Compared with the PSD group, the Cil group had significantly decreased RhoA protein (F_2, 6_ = 13.280, p = 0.0220), RhoA mRNA (F_2, 6_ = 18.000, p = 0.0110), and RhoA-GTP (F_2, 6_ = 28.000, p = 0.0009) levels. No significant differences were observed between the Cil and Con group in RhoA protein (F_2, 6_ = 13.280, p = 0.5093), RhoA mRNA (F_2, 6_ = 18.000, p = 0.4193), and RhoA-GTP (F_2, 6_ = 28.000, p = 0.2604) levels.

The elevated levels of pro-inflammatory cytokines, TNF-α and IL-1β, and the pro-inflammatory signaling activator, NF-κB p65, were used as molecular evidence for microglial activation. The levels of these factors in the hippocampus in each mice group is presented in Figures 4H–M. The PSD group had significantly higher TNF-α protein (F_2, 6_ = 6.462, p = 0.0491), TNF-α mRNA (F_2, 6_ = 83.100, p < 0.0001), IL-1β protein (F_2, 6_ = 19.360, p = 0.0056), and IL-1β mRNA (F_2, 6_ = 68.240, p = 0.0001) and NF-κB p65 subunit (F_2, 6_ = 29.810, p = 0.0043) levels than the Con group. The Cil group had significantly lower TNF-α protein (F_2, 6_ = 6.462, p = 0.0456), TNF-α mRNA (F_2, 6_ = 83.100, p = 0.0001), IL-1β protein (F_2, 6_ = 19.360, p = 0.0031), IL-1β mRNA (F_2, 6_ = 68.240, p = 0.0002), and NF-κB p65 subunit (F_2, 6_ = 29.810, p = 0.0007) levels than the PSD group. No significant differences were observed between the Cil and Con group in TNF-α protein (F_2, 6_ = 6.462, p = 0.9980), TNF-α mRNA (F_2, 6_ = 83.100, p < 0.2782), IL-1β protein (F_2, 6_ = 19.360, p = 0.8103), and IL-1β mRNA (F_2, 6_ = 68.240, p = 6,186) and NF-κB p65 subunit (F_2, 6_ = 29.810, p = 0.1564) levels.

These results indicate that cilostazol can inhibit the activation of the RhoA/NF-κB signaling pathway and the expression of pro-inflammatory cytokines in the hippocampus of CUMS-subjected IS mice.

### 3.5 Cilostazol inhibits RhoA/NF-κB signaling pathway activation in BV2 microglia to reduce pro-inflammatory cytokines expression and rescue apoptosis in primary hippocampal neurons

In the aforementioned animal experiments, cilostazol regulated neuronal apoptosis, microglial activation, RhoA/NF-κB signaling pathway activation and pro-inflammatory cytokines expression, suggesting that the involvement of these factors in cilostazol’s PSD prevention mechanism. However, owing to the complex physiological responses in the animals, these experiments could not conclusively demonstrate cilostazol’s direct targeting effect on neurons or clearly reveal the cilostazol-induced downstream RhoA/NF-κB signaling pathway changes in the microglia. Therefore, a series of experiments was conducted using BV2 microglia and primary hippocampal neurons to better understand the temporal sequence of these events and to fully elucidate the cilostazol’s PSD prevention mechanisms.

#### 3.5.1 RhoA activation in BV2 microglia promotes NF-κB activation and pro-inflammatory cytokines secretion

**FIGURE 5:**
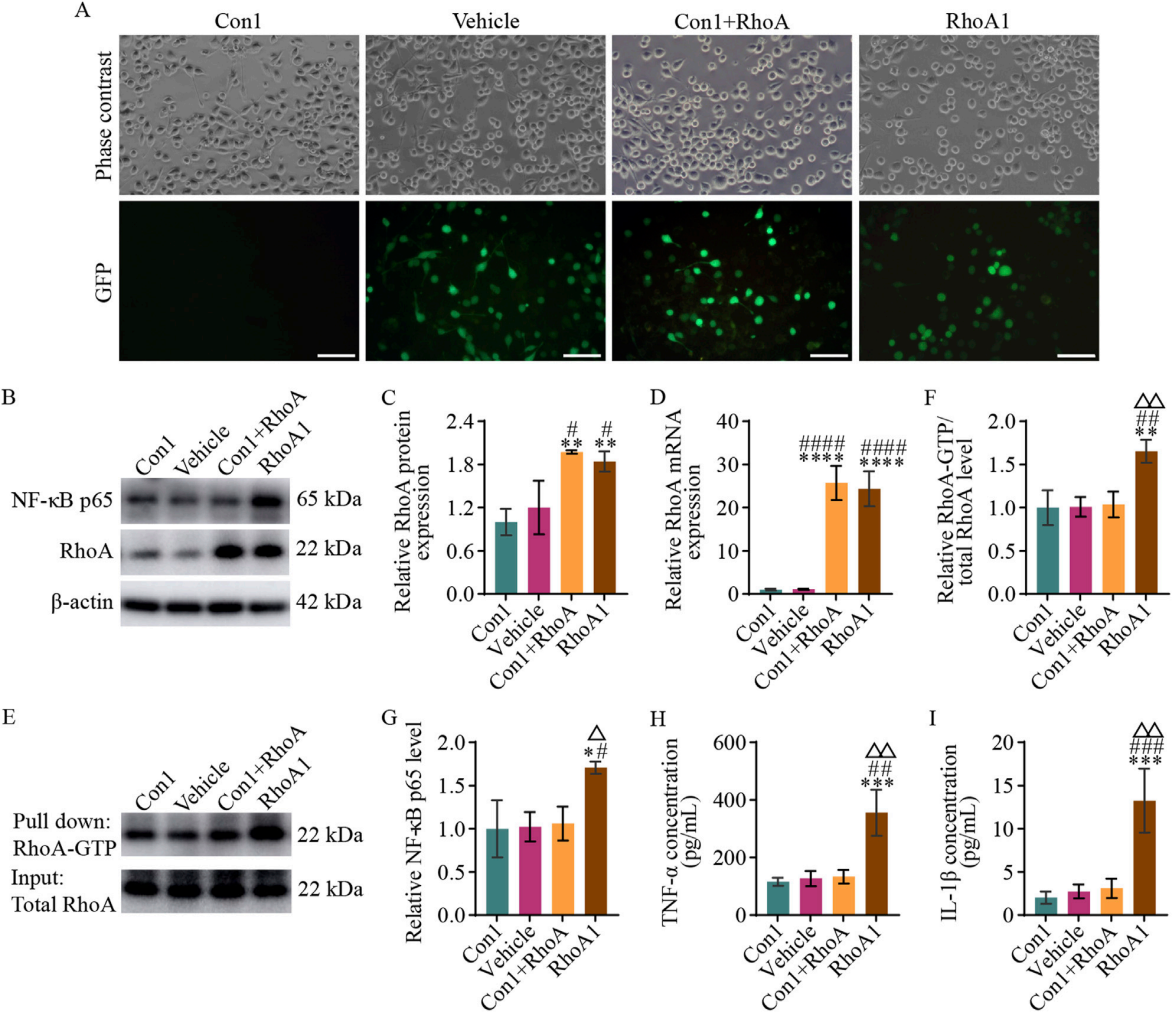
RhoA activation in BV2 microglia promotes NF-κB activation and pro-inflammatory cytokines secretion. **(A)** Representative photomicrographs (scale bars: 100 μm) in the different groups of BV2 microglia. The Con1 group is the normal BV2 microglia; Vehicle group is the BV2 microglia with empty vector transfection; Con1+RhoA group is the BV2 microglia with overexpressing RhoA; and RhoA1 group is the BV2 microglia with the overexpressing and activated RhoA. **(B)** Representative blots exhibiting RhoA and NF-κB p65 protein relative expression levels in the different groups. **(C,D)** Analysis of RhoA protein **(C)** and mRNA **(D)** relative expression levels in the different groups. **(E)** Representative blots exhibiting the relative RhoA activation levels in the different groups. **(F)** Analysis of RhoA relative activation levels in the different groups. **(G)** Analysis of NF-κB p65 relative expression levels in the different groups. **(H,I)** Analysis of TNF-α **(H)** and IL-1β **(I)** relative secretion levels in the culture medium in the different groups. Compared with the Con1 group, *p < 0.05, **p < 0.01, ***p < 0.001, ****p < 0.0001; compared with the Vehicle group, #p < 0.05, ##p < 0.01, ###p < 0.001, ####p < 0.0001; compared with the Con1+RhoA group, △p < 0.05, △△p < 0.01.

According to the protocol described in Section 2.6, four groups of a BV2 microglia model with stable RhoA overexpression or activation were established: Con1 (normal BV2 microglia), Vehicle (BV2 microglia with empty vector transfection), Con1+RhoA (BV2 microglia with overexpression RhoA), and RhoA1 (BV2 microglia with overexpressed and activated RhoA) groups were prepared (Figure 5A).

Western blot and qPCR results (Figures 5B–D) revealed no significant difference in RhoA protein (F_3, 8_ = 14.210, p = 0.6781) and RhoA mRNA (F_3, 8_ = 71.190, p > 0.9999) expression levels between the Con1 and Vehicle groups. However, RhoA protein expression levels were significantly higher in the Con1+RhoA and RhoA1 groups than in the Con1 and Vehicle groups (Con1 vs. Con1+RhoA, F_3, 8_ = 14.210, p = 0.0027; Vehicle vs. Con1+RhoA, F_3, 8_ = 14.210, p = 0.0109; Con1 vs. RhoA1, F_3, 8_ = 14.210, p = 0.0066; Vehicle vs. RhoA1, F_3, 8_ = 14.210, p = 0.0297), and RhoA mRNA expression levels were significantly higher in the Con1+RhoA and RhoA1 groups as well, than in the Con1 and Vehicle groups (Con1 vs. Con1+RhoA, F_3, 8_ = 71.190, p < 0.0001; Vehicle vs. Con1+RhoA, F_3, 8_ = 71.190, p < 0.0001; Con1 vs. RhoA1, F_3, 8_ = 71.190, p < 0.0001; Vehicle vs. RhoA1, F_3, 8_ = 71.190, p < 0.0001). These findings confirmed RhoA overexpression in the Con1+RhoA and RhoA1 groups, as expected.

The Pull-down assay results (Figures 5E,F) revealed no significant difference in RhoA-GTP levels among the Con1, Vehicle, and Con1+RhoA groups (Con1 vs. Vehicle, F_3, 8_ = 12.910, p = 0.9998; Con1 vs. Con1+RhoA, F_3, 8_ = 12.910, p = 0.9900; Vehicle vs. Con1+RhoA, F_3, 8_ = 12.910, p = 0.9963). However, the RhoA1 group had significantly higher RhoA-GTP levels than the Con1 (F_3, 8_ = 12.910, p = 0.0036), Vehicle (F_3, 8_ = 12.910, p = 0.0040) and Con1+RhoA (F_3, 8_ = 12.910, p = 0.0052) groups. These findings indicated that the Con1+RhoA group had high RhoA expression with normal activation, whereas the RhoA1 group had both high RhoA expression and activation.

Western blot, qPCR and ELISA results (Figures 5B,G–I) revealed no significant differences in NF-κB p65 subunit (Con1 vs. Vehicle, F_3, 8_ = 7.625, p = 0.9991; Con1 vs. Con1+RhoA, F_3, 8_ = 7.625, p = 0.9845; Vehicle vs. Con1+RhoA, F_3, 8_ = 7.625, p = 0.9960) and TNF-α (Con1 vs. Vehicle, F_3, 8_ = 20.210, p = 0.9983; Con1 vs. Con1+RhoA, F_3, 8_ = 20.210, p = 0.9618; Vehicle vs. Con1+RhoA, F_3, 8_ = 20.210, p = 0.9983) and IL-1β (Con1 vs. Vehicle, F_3, 8_ = 21.220, p = 0.9705; Con1 vs. Con1+RhoA, F_3, 8_ = 21.220, p = 0.9092; Vehicle vs. Con1+RhoA, F_3, 8_ = 21.220, p = 0.9957) levels among the Con1, Vehicle and Con1+RhoA groups. However, the RhoA1 group exhibited a significantly higher NF-κB p65 (RhoA1 vs. Con1, F_3, 8_ = 7.625, p = 0.0155; RhoA1 vs. Vehicle, F_3, 8_ = 7.625, p = 0.0185; RhoA1 vs. Con1+RhoA, F_3, 8_ = 7.625, p = 0.0248), TNF-α (RhoA1 vs. Con1, F_3, 8_ = 20.210, p = 0.0008; RhoA1 vs. Vehicle, F_3, 8_ = 20.210, p = 0.0011; RhoA1 vs. Con1+RhoA, F_3, 8_ = 20.210, p = 0.0013), and IL-1β (RhoA1 vs. Con1, F_3, 8_ = 21.220, p = 0.0006; RhoA1 vs. Vehicle, F_3, 8_ = 21.220, p = 0.0009; RhoA1 vs. Con1+RhoA, F_3, 8_ = 21.220, p = 0.0012) levels than the Con1, Vehicle and Con1+RhoA groups. These findings suggested that RhoA activation in BV2 microglia drives NF-κB signaling pathway activation and pro-inflammatory cytokines secretion.

Since the Vehicle and the Con1 group did not exhibit any-significant differences in RhoA protein (F_3, 8_ = 14.210, p = 0.6781), RhoA mRNA (F_3, 8_ = 71.190, p > 0.9999), NF-κB p65 subunit (F_3, 8_ = 7.625, p = 0.9991), RhoA-GTP (F_3, 8_ = 12.910, p = 0.9998), TNF-α (F_3, 8_ = 20.210, p = 0.9883), and IL-1β (F_3, 8_ = 21.220, p = 0.9705) (Figures 5B–I) levels, the Vehicle group was not included in subsequent experiments.

#### 3.5.2 Cilostazol inhibits RhoA/NF-κB signaling pathway activation and pro-inflammatory cytokines secretion in BV2 microglia

**FIGURE 6:**
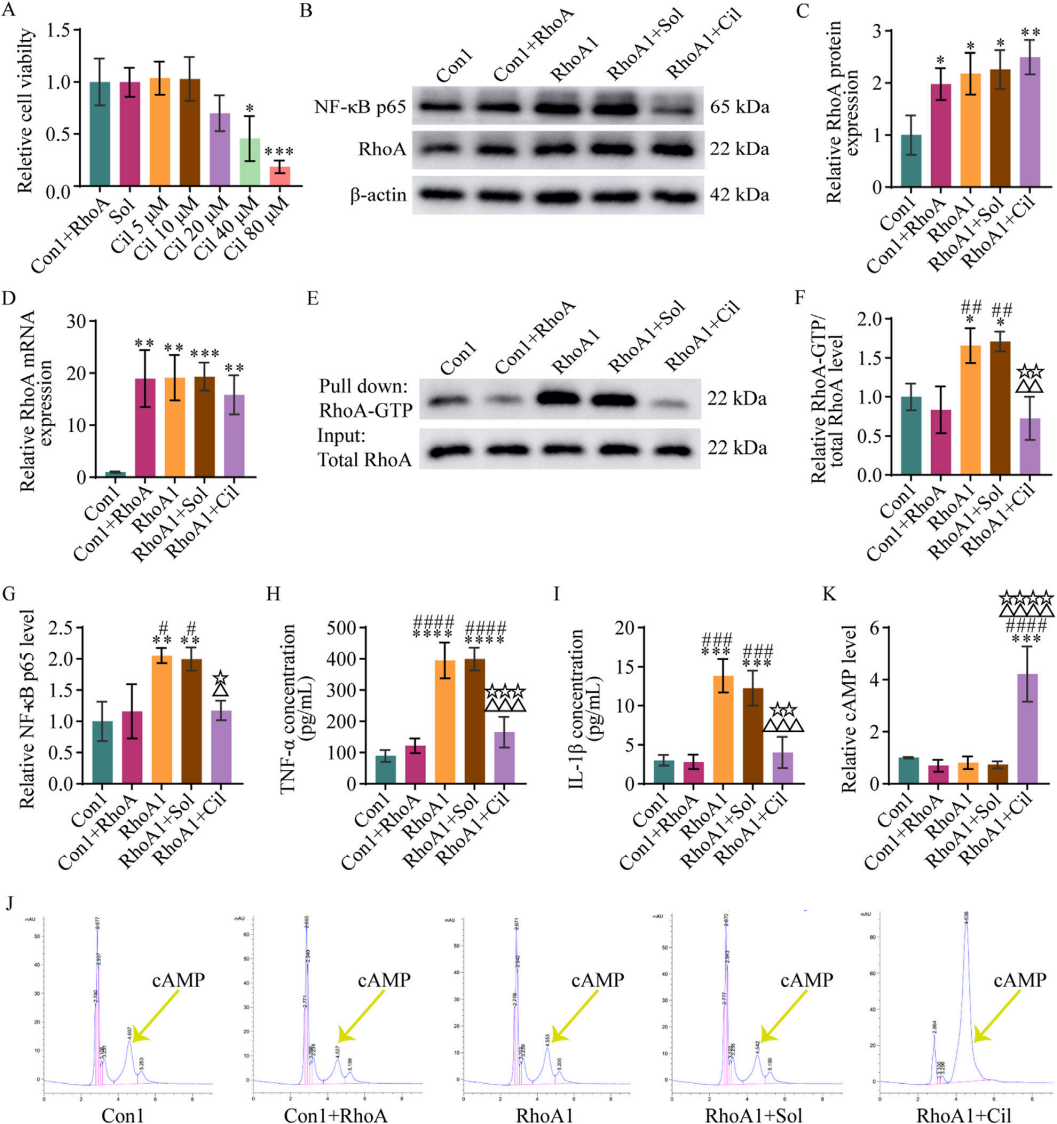
Cilostazol inhibits RhoA/NF-κB signaling pathway activation and pro-inflammatory cytokine secretion in BV2 microglia. **(A)** Analysis of different doses of cilostazol on the viability of BV2 microglia with RhoA overexpression. The Con1+RhoA group is the normal BV2 microglia with RhoA overexpression. The Sol, Cil 5 μM, Cil 10 μM, Cil 20 μM, Cil 40 μM, and Cil 80 μM groups are the RhoA-overexpressing BV2 microglia treated with 0, 5, 10, 20, 40, or 80 μM cilostazol, respectively, dissolved in dimethyl sulfoxide (DMSO). **(B)** Representative blots exhibiting RhoA and NF-κB p65 protein relative expression levels in the different groups of BV2 microglia. The Con1 group is the normal BV2 microglia; Vehicle group is the BV2 microglia with empty vector transfection; RhoA1 group is the BV2 microglia with overexpressing and activated RhoA; RhoA1+Sol group is the BV2 microglia with overexpressing and activated RhoA treated with DMSO; and RhoA1+Cil group is the BV2 microglia with overexpressing and activated RhoA treated with 10 μM cilostazol. **(C,D)** Analysis of RhoA protein **(C)** and mRNA **(D)** relative expression levels in the different groups. **(E)** Representative blots exhibiting RhoA relative activation levels in the different groups. **(F)** Analysis of RhoA-GTP relative levels in the different groups. **(G)** Analysis of NF-κB p65 relative levels in the different groups. **(H,I)** Analysis of TNF-α **(H)** and IL-1β **(I)** relative levels in the culture medium in the different groups. **(J)** Representative peak area graphs exhibiting cAMP relative levels in the different groups. **(K)** Analysis of cAMP relative levels in the different groups. One-way ANOVA followed by Tukey’s *post hoc* multiple comparison test used to compare the results between groups, and expressed as mean ± standard deviation. *P* < 0.05 was considered to be statistically significant (Compared with the Con1 group, *p < 0.05, **p < 0.01, ***p < 0.001, ****p < 0.0001; compared with the Con1+RhoA group, #p < 0.05, ##p < 0.01, ###p < 0.001, ####p < 0.0001; compared with the RhoA1 group, △p < 0.05, △△p < 0.01, △△△p < 0.001, △△△△p < 0.0001; compared with the RhoA1+Sol group, ☆p < 0.05, ☆☆p < 0.01, ☆☆☆p < 0.001, ☆☆☆☆p < 0.0001).

To investigate whether cilostazol could regulate the RhoA/NF-κB signaling pathway activation, a CCK-8 assay was used to determine the effect of different doses of cilostazol on the viability of BV2 microglia with RhoA overexpression. The results indicated that 10 μM cilostazol was the optimal dose (Figure 6A).

Next, BV2 microglia with activated RhoA were treated with 10 μM cilostazol, and the regulatory effect of cilostazol on the RhoA/NF-κB signaling pathway activation and pro-inflammatory cytokines secretion were examined using Western blot, qPCR, and pull-down assays. The results (Figures 6B–D) revealed that RhoA protein and mRNA expression were significantly higher in the RhoA1+Cil group than in the Con1 group (F_4, 10_ = 7.791, p = 0.0033; F_4, 10_ = 13.260, p = 0.0047), albeit comparable with those in the RhoA1 group (F_4, 10_ = 7.791, p = 0.8104; F_4, 10_ = 13.260, p = 0.8109), suggesting that cilostazol treatment did not significantly affect the increased expression of RhoA induced by the RhoA-overexpressing vector in BV2 microglia. However, the RhoA-GTP levels in the RhoA1+Cil group were significantly lower than those in the RhoA1 group (F_4, 10_ = 12.440, p = 0.0038) and not significantly different from those in the Con1 (F_4, 10_ = 12.440, p = 0.5979) or Con1+RhoA groups (F_4, 10_ = 12.440, p = 0.9737) (Figures 6E,F), indicating that cilostazol effectively inhibited RhoA activation in BV2 microglia. Additionally, NF-κB p65 subunit, TNF-α, and IL-1β levels in the RhoA1+Cil group were significantly lower than those in the RhoA1 group (F_4, 10_ = 10.560, p = 0.0166; F_4, 10_ = 43.300, p = 0.0003; F_4, 10_ = 29.490, p = 0.0003) and not significantly different from those in the Con1 (F_4, 10_ = 10.560, p = 0.9286; F_4, 10_ = 43.300, p = 0.2115; F_4, 10_ = 29.490, p = 0.9454) or Con1+RhoA groups (F_4, 10_ = 10.560, p > 0.9999; F_4, 10_ = 43.300, p = 0.6792; F_4, 10_ = 29.490, p = 0.9051) (Figures 6G–I), suggesting that cilostazol inhibited RhoA/NF-κB signaling pathway activation and TNF-α and IL-1β secretion in BV2 microglia.

cAMP inhibits RhoA activation (Aburima et al., 2013; Gündüz et al., 2019; Fan et al., 2020; Li L. et al., 2021; Balraj et al., 2024), and cilostazol inhibits of cAMP degradation (Park et al., 2013; Kherallah et al., 2022). The results of the HPLC-based exploration of cilostazol’s RhoA activation inhibitory mechanism to detect the drug’s effect on cAMP levels in BV2 microglia (Figures 6J,K) revealed no significant difference in cAMP levels among the Con1, Con1+RhoA, RhoA1 and RhoA1+Sol groups (Con1 vs. Con1+RhoA, F_4, 10_ = 27.850, p = 0.9412; Con1 vs. RhoA1, F_4, 10_ = 27.850, p = 0.9893; Con1 vs. RhoA1+Sol, F_4, 10_ = 27.850, p = 0.9610). However, the cAMP levels in the RhoA1+Cil group were significantly higher than those in the other groups (RhoA1+Cil vs. Con1, F_4, 10_ = 27.850, p = 0.0001; RhoA1+Cil vs. Con1+RhoA, F_4, 10_ = 27.850, p < 0.0001; RhoA1+Cil vs. RhoA1, F_4, 10_ = 27.850, p < 0.0001; RhoA1+Cil vs. RhoA1+Sol, F_4, 10_ = 27.850, p < 0.0001). These findings suggest that cilostazol inhibits RhoA/NF-κB signaling pathway activation and TNF-α and IL-1β secretion by increasing cAMP levels in BV2 microglia.

The results of the evaluation of DMSO interference with t RhoA/NF-κB signaling pathway activation and TNF-α and IL-1β secretion revealed no significant differences in RhoA protein, RhoA mRNA, RhoA-GTP, NF-κB p65, TNF-α, IL-1β, and cAMP levels between the RhoA1+Sol and RhoA1 groups (F_4, 10_ = 7.791, p = 0.9986; F_4, 10_ = 13.260, p = 0.9999; F_4, 10_ = 12.440, p = 0.9985; F_4, 10_ = 10.560, p = 0.9989; F_4, 10_ = 43.300, p > 0.9999; F_4, 10_ = 29.490, p = 0.7924; F_4, 10_ = 27.850, p = 0.9996) (Figures 6B–K). However, significant differences were observed in RhoA-GTP, NF-κB p65, TNF-α, IL-1β, and cAMP levels between the RhoA1+Sol and RhoA1+Cil groups (F_4, 10_ = 12.440, p = 0.0026; F_4, 10_ = 10.560, p = 0.0247; F_4, 10_ = 43.300, p = 0.0002; F_4, 10_ = 29.490, p = 0.0012; F_4, 10_ = 27.850, p < 0.0001) (Figures 6E–K). These findings indicate that DMSO did not significantly interfere with RhoA expression, RhoA/NF-κB signaling pathway activation, cAMP levels and TNF-α and IL-1β secretion in BV2 microglia. Therefore, changes in RhoA-GTP, NF-κB p65, TNF-α, IL-1β, and cAMP levels were solely attributed to cilostazol, consequently, the RhoA1+Sol group was not included in subsequent experiments involving the primary hippocampal neurons.

#### 3.5.3 Cilostazol mitigates primary hippocampal neuronal apoptosis induced by RhoA-activated BV2 microglia

**FIGURE 7:**
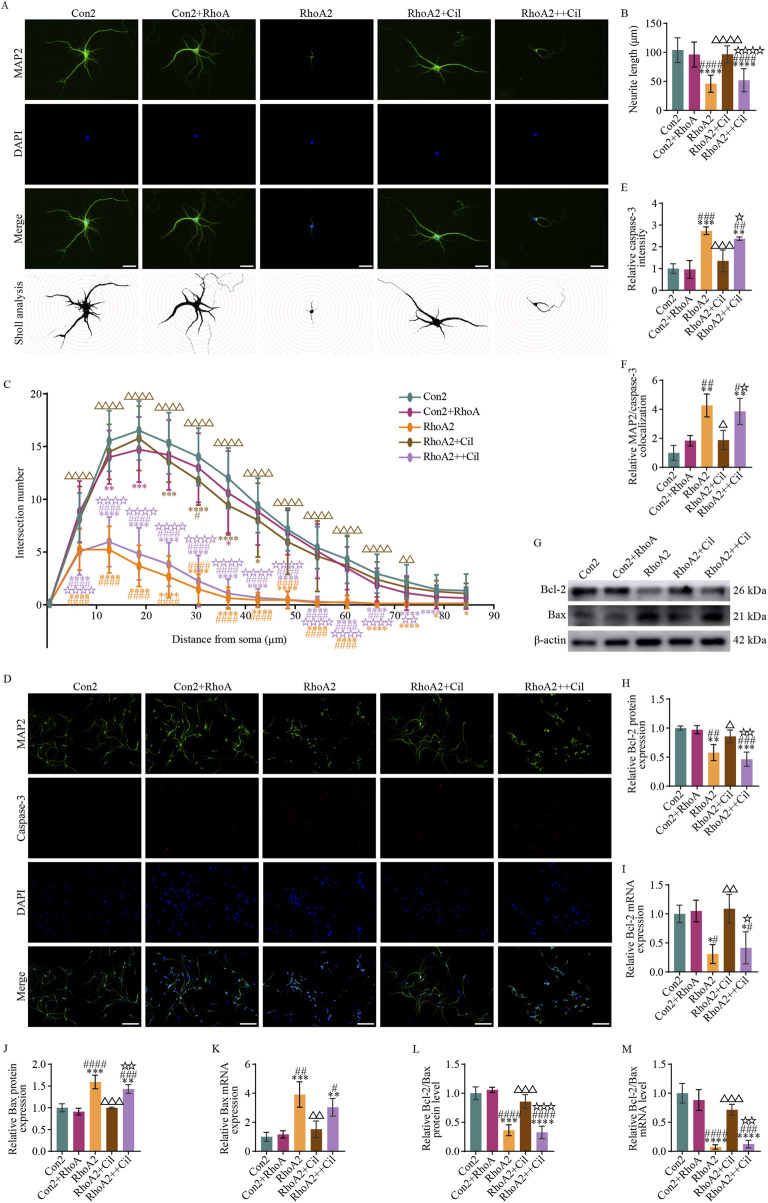
Cilostazol mitigates primary hippocampal neuronal apoptosis induced by RhoA-activated BV2 microglia. **(A)** Representative photomicrographs of immunofluorescence labeling (scale bars: 20 μm) and intersections between the dendrites and concentric circles (with radii increasing by 100 pixels, approximately 6 μm) in the different groups of primary hippocampal neurons. MAP2, a marker for dendrites, appears green and depicts the distribution of neurons. DAPI, a fluorescent dye that binds to cell nuclei, appears blue and depicts the distribution of all cultured cells. The “Merge” image combines both fluorescence types into a composite image, depicting the proportion of neurons. The Con2 group is the primary hippocampal neurons cultured in the complete primary neuron culture medium. The Con2+RhoA group is the primary hippocampal neurons cultured in 50% complete primary neuron culture medium and 50% culture supernatant from the RhoA-overexpressing BV2 microglia. The RhoA2 group is the primary hippocampal neurons cultured in 50% complete primary neuron culture medium and 50% culture supernatant from the RhoA-activated BV2 microglia. The RhoA2+Cil group is the primary hippocampal neurons cultured in 50% complete primary neuron culture medium and 50% culture supernatant from the RhoA-activated BV2 microglia treated with 10 μM cilostazol for 12 h. The RhoA2++Cil group is the primary hippocampal neurons cultured in 50% complete primary neuron culture medium and 50% culture supernatant from the RhoA-activated BV2 microglia and treated with 10 μM cilostazol. **(B)** Analysis of dendrite length in the different groups of primary hippocampal neurons (n = 30). **(C)** Analysis of intersection numbers between the dendrites and concentric circles in the different groups (n = 30). **(D)** Representative photomicrographs of immunofluorescence labeling (scale bars: 50 μm) in the different groups. Caspase-3, a marker for apoptotic cells, appears red and depicts the distribution of apoptosis neurons. The “Merge” image combines three fluorescence types into a composite image, depicting the proportion of apoptosis neurons. **(E,F)** Analysis of caspase-3 fluorescence intensity **(E)** and its colocalization with MAP-2 **(F)**. **(G)** Representative blots exhibiting Bcl-2 and Bax protein relative expression levels in the different groups. **(H–I)** Analysis of Bcl-2 protein **(H)**, Bcl-2 mRNA **(I)**, Bax protein **(J)** and Bax mRNA **(K)** relative expression levels in the different groups. **(L,M)** Analysis of the Bcl-2/Bax ratio at protein **(L)** and mRNA **(M)** levels in the different groups. Compared with the Con2 group, *p < 0.05, **p < 0.01, ***p < 0.001, ****p < 0.0001; compared with the Con2+RhoA group, #p < 0.05, ##p < 0.01, ###p < 0.001, ####p < 0.0001; compared with the RhoA2 group, △p < 0.05, △△p < 0.01, △△△p < 0.001, △△△△p < 0.0001; compared with the RhoA2+Cil group, ☆p < 0.05, ☆☆p < 0.01, ☆☆☆p < 0.001, ☆☆☆☆p < 0.0001.

According to the protocol described in Section 2.8, the effects of cilostazol-treated BV2 microglia with RhoA overexpression and activation on the survival of these primary hippocampal neurons were explored. The results indicated nearly no significant differences in dendritic length (Figures 7A,B) (F_4, 145_ = 64.980, p = 0.5276), the number of dendritic intersections with most concentric circles (Figures 7A,C) (second, F_84, 3190_ = 58.760, p = 0.6473; third, F_84, 3190_ = 58.760, p = 0.0033; fourth, F_84, 3190_ = 58.760, p = 0.0003; fifth, F_84, 3190_ = 58.760, p = 0.0885; sixth, F_84, 3190_ = 58.760, p = 0.1271; seventh, F_84, 3190_ = 58.760, p = 0.0123; eighth, F_84, 3190_ = 58.760, p = 0.6473; ninth, F_84, 3190_ = 58.760, p = 0.9598; 10th, F_84, 3190_ = 58.760, p = 0.9417; 11th, F_84, 3190_ = 58.760, p = 0.3163; 12th, F_84, 3190_ = 58.760, p = 0.1064; 13th, F_84, 3190_ = 58.760, p = 0.0598; 14th, F_84, 3190_ = 58.760, p = 0.4504; 15th, F_84, 3190_ = 58.760, p = 0.4989), average caspase-3 fluorescence intensity (Figures 7D,E) (F_4, 10_ = 19.920, p = 0.9998) and its colocalization with neurons (Figures 7D,F) (F_4, 10_ = 13.210, p = 0.5703), Bcl-2 protein (Figures 7G,H) (F_4, 10_ = 16.600, p = 0.9968), Bcl-2 mRNA (Figure 7I) (F_4, 10_ = 9.582, p = 0.9981), Bax protein (Figures 7G,J) (F_4, 10_ = 28.450, p = 0.8132), and Bax mRNA (Figure 7K) (F_4, 10_ = 15.320, p = 0.9954) relative expression levels, and Bcl-2/Bax ratio at both the protein (Figure 7L) (F_4, 10_ = 37.380, p = 0.9450) and mRNA (Figure 7M) (F_4, 10_ = 37.290, p = 0.7736) levels between the Con2 and Con2+RhoA groups. These findings suggest that the conditioned medium from the RhoA-overexpressed BV2 microglia does not affect the survival of primary hippocampal neurons.

Conversely, compared with the Con2 group, the RhoA2 group had a significantly decreased dendritic length (Figures 7A,B) (F_4, 145_ = 64.980, p < 0.0001), marked decreased number of dendritic intersections with concentric circles (Figures 7A,C) (2nd–13th, F_84, 3190_ = 58.760, p < 0.0001; 14th, F_84, 3190_ = 58.760, p = 0.0157; 15th, F_84, 3190_ = 58.760, p = 0.0394), notably increased average caspase-3 fluorescence intensity (Figures 7D,E) (F_4, 10_ = 19.920, p = 0.0004) and its colocalization with neurons (Figures 7D,F) (F_4, 10_ = 13.210, p = 0.0011), decreased Bcl-2 protein (Figures 7G,H) (F_4, 10_ = 16.600, p = 0.0035) and Bcl-2 mRNA (Figure 7I) (F_4, 10_ = 9.582, p = 0.0166) relative expression levels, increased Bax protein (Figures 7G,J) (F_4, 10_ = 28.450, p = 0.0002) and Bax mRNA (Figure 7K) (F_4, 10_ = 15.320, p = 0.0007) relative expression levels, and decreased Bcl-2/Bax ratio at both the protein (Figure 7L) (F_4, 10_ = 37.380, p = 0.0001) and mRNA (Figure 7M) (F_4, 10_ = 37.290, p < 0.0001) levels. These findings suggest that the conditioned medium from the RhoA-activated BV2 microglia restricts dendritic growth and induces apoptosis in primary hippocampal neurons.

Almost no significant differences were observed between the RhoA2+Cil and Con2 groups in dendrite length (Figures 7A,B) (F_4, 145_ = 64.980, p = 0.5580), the number of dendritic intersections with most concentric circles (Figures 7A,C) (second, F_84, 3190_ = 58.760, p = 0.9994; third, F_84, 3190_ = 58.760, p = 0.1271; fourth, F_84, 3190_ = 58.760, p = 0.4034; fifth, F_84, 3190_ = 58.760, p = 0.0010; sixth, F_84, 3190_ = 58.760, p < 0.0001; seventh, F_84, 3190_ = 58.760, p < 0.0001; eighth, F_84, 3190_ = 58.760, p = 0.0123; ninth, F_84, 3190_ = 58.760, p = 0.0598; 10th, F_84, 3190_ = 58.760, p = 0.3586; 11th, F_84, 3190_ = 58.760, p = 0.5981; 12th, F_84, 3190_ = 58.760, p = 0.7413; 13th, F_84, 3190_ = 58.760, p = 0.6473; 14th, F_84, 3190_ = 58.760, p = 0.9911; 15th, F_84, 3190_ = 58.760, p = 0.9738), average caspase-3 fluorescence intensity (Figures 7D,E) (F_4, 10_ = 19.920, p = 0.6612) and its colocalization with neurons (Figures 7D,F) (F_4, 10_ = 13.210, p = 0.5275), Bcl-2 protein (Figures 7G,H) (F_4, 10_ = 16.600, p = 0.4757), Bcl-2 mRNA (Figure 7I) (F_4, 10_ = 9.582, p = 0.9841), Bax protein (Figures 7G,J) (F_4, 10_ = 28.450, p > 0.9999), and Bax mRNA (Figure 7K) (F_4, 10_ = 15.320, p = 0.7920) relative expression levels, and the Bcl-2/Bax ratio at both the protein (Figure 7L) (F_4, 10_ = 37.380, p = 0.4412) and mRNA (Figure 7M) (F_4, 10_ = 37.290, p = 0.0989) levels. These findings suggest that the conditioned medium from the cilostazol-treated BV2 microglia with activated RhoA does not affect the survival of primary hippocampal neurons.

Additionally, a comparison of the RhoA2+Cil and RhoA2 groups revealed a significantly increased dendritic length (Figures 7A,B) (F_4, 145_ = 64.980, p < 0.0001), increased number of dendrite intersections with most concentric circles (Figures 7A,C) (2nd–12th, F_84, 3190_ = 58.760, p < 0.0001; 13th, F_84, 3190_ = 58.760, p = 0.0033; 14th, F_84, 3190_ = 58.760, p = 0.0598; 15th, F_84, 3190_ = 58.760, p = 0.1775), significantly decreased average caspase-3 fluorescence intensity (Figures 7D,E) (F_4, 10_ = 19.920, p = 0.0024) and its colocalization with neurons (Figures 7D,F) (F_4, 10_ = 13.210, p = 0.0101), increase Bcl-2 protein (Figures 7G,H) (F_4, 10_ = 16.600, p = 0.0451) and Bcl-2 mRNA (Figure 7I) (F_4, 10_ = 9.582, p = 0.0077) relative expression levels, decreased Bax protein (Figures 7G,J) (F_4, 10_ = 28.450, p = 0.0002) and Bax mRNA (Figure 7K) (F_4, 10_ = 15.320, p = 0.0030) relative expression levels, and increased Bcl-2/Bax ratio at both the protein (Figure 7L) (F_4, 10_ = 37.380, p = 0.0009) and mRNA (Figure 7M) (F_4, 10_ = 37.290, p = 0.0006) levels. These findings suggest that cilostazol intervention can reverse the dendritic growth restriction and neuronal apoptosis induced by the conditioned medium from the RhoA-activated BV2 microglia.

Furthermore, compared with the Con2 group, the RhoA2++Cil group exhibited a significantly decreased dendritic length (Figures 7A,B) (F_4, 145_ = 64.980, p < 0.0001), decreased number of dendritic intersections with most concentric circles (Figures 7A,C) (2nd–13th, F_84, 3190_ = 58.760, p < 0.0001; 14th, F_84, 3190_ = 58.760, p < 0.0200; 15th, F_84, 3190_ = 58.760, p = 0.0598), increased average caspase-3 fluorescence intensity (Figures 7D,E) (F_4, 10_ = 19.920, p = 0.0025) and its colocalization with neurons (Figures 7D,F) (F_4, 10_ = 13.210, p = 0.0029), and significantly decreased Bcl-2 protein (Figures 7G,H) (F_4, 10_ = 16.600, p = 0.0006) and Bcl-2 mRNA (Figure 7I) (*F*_4, 10_ = 9.582, p = 0.0434) relative expression levels, increased Bax protein (Figures 7G,J) (F_4, 10_ = 28.450, p = 0.0024) and Bax mRNA (Figure 7K) (F_4, 10_ = 15.320, p = 0.0091) relative expression levels, and decreased Bcl-2/Bax ratio at both the protein (Figure 7L) (F_4, 10_ = 37.380, p < 0.0001) and mRNA (Figure 7M) (F_4, 10_ = 37.290, p < 0.0001) levels. These findings suggest that direct treatment of primary hippocampal neurons with cilostazol could not reverse the dendritic growth restriction and neuronal apoptosis induced by the conditioned medium from the RhoA-activated BV2 microglia.

Collectively, these results indicate that cilostazol prevents the growth restriction and apoptosis of primary hippocampal neurons by inhibiting pro-inflammatory cytokines secretion from RhoA-activated BV2 microglia.

## 4 Discussion

Ischemic stroke is the most common cerebrovascular disease (Feske, 2021), and PSD complicates IS treatment, challenging successful recovery. Consequently, PSD prevention has garnered increasing attention. Cilostazol was initially reported to have positive therapeutic effects in one Japanese patient with PSD and seven older patients with MDD unresponsive to antidepressants; however, this finding did not initially garner widespread attention (Baba et al., 2007; Nishimura et al., 2007). The use of cilostazol as an adjunctive treatment for MDD was reported to have significant antidepressant effects (Khadivi et al., 2022). This further suggests that cilostazol may hold considerable therapeutic potential for certain patients with depression. In this study, we used CUMS-subjected IS mice to further investigate the efficacy of cilostazol in preventing PSD and its underlying signaling pathways.

The SPT, OFT, TST and MWMT were used to assess the preventive effects of cilostazol on PSD. The SPT results revealed that cilostazol effectively prevented the decrease in sucrose preference, suggesting protection of the pleasure-related centers in the brain. The OFT results indicated that cilostazol significantly increased the distance traveled by mice in the central area of the open field, demonstrating that it could prevent depressive behaviors, such as reduced spontaneous exploratory activity and social withdrawal. The TST results revealed that cilostazol significantly reduced the immobility time in suspended mice, confirming that it could improve despair-related behaviors. The MWMT results demonstrated that cilostazol significantly shortened the latency time for mice to find the escape platform, and increased the percentage of distance in the target quadrant, average speed, activity time on the platform and number of platform crossings, suggesting its contribution to cognitive impairment amelioration and promotion of more purposeful, goal-directed behavior to locate the escape platform. Cilostazol was reported to significantly improve cognition and emotional behavior in diabetic dementia mice (Hishikawa et al., 2017), and regulate excitatory movement, social failures, repetitive behaviors, and anxiety in autism mice (Luhach et al., 2021). Similar findings in other studies (Hishikawa et al., 2017; Luhach et al., 2021; Ravi et al., 2025), along with the behavioral test results from the present study (Section 3.1), suggest that cilostazol may be a promising treatment for preventing PSD.

Understanding the antidepressant mechanism of cilostazol is crucial for advancing its clinical use in preventing PSD onset. A study of RNA sequencing of peripheral blood mononuclear-macrophages from cilostazol-treated humans revealed a significant downregulation *MRC1*, *CXCL11*, and *CXCL10*. The downregulation of *MRC1*, primarily expressed in activated macrophage subpopulations, suggests that more macrophages may be transitioning to a resting state. The downregulation of chemokines, *CXCL10* and *CXCL11*, would reduce the recruitment of immune cells, including M1 macrophages, to areas with thrombotic or hemorrhagic plaques. These findings suggest that cilostazol inhibits the transformation of monocytes into pro-inflammatory macrophages, helping reduce inflammation (Fan et al., 2024). In hypercholesterolemia rats, cilostazol reportedly exerted anti-inflammatory effects by inhibiting the PLC/PKC-α/p38/IκB-α/NF-κB signaling pathway (Brazão et al., 2023), thereby alleviating hypercholesterolemia-induced inflammatory cardiac damage (de Oliveira Lopes et al., 2022). Cilostazol reportedly prevented the development of anxiety symptoms and post-traumatic stress disorder (PTSD)-induced increases in hippocampal IDO and IL-1β, improving neuroinflammation, and has been proposed as a promising candidate for further PTSD pharmacotherapeutic research (Sadeghi et al., 2024). Cilostazol has been demonstrated to reduce the NF-κB p65-evoked inflammatory response and astrocyte activation via the AMPK/SIRT1 signaling pathway; promote Nrf2 antioxidant effect; reduce and increase the expression level of Bax and Bcl2 protein, respectively; restore the BDNF/TrkB/CREB neuroprotective axis function; and restore mitochondrial dysfunction in brain cells, thereby alleviating liver disease-induced nerve damage (Gad et al., 2025). Moreover, cilostazol can reportedly alleviate acute stroke-induced cognitive impairment by decreasing hippocampal neuronal apoptosis (Anastasiadou et al., 2025). Furthermore, studies on cilostazol’s neuroprotective mechanisms in rodent models of anxiety, transient cerebral ischemia, autism spectrum disorder, Huntington’s disease, and chronic cerebral ischemia have revealed its modulation of inflammation and apoptotic signaling pathways (El-Abhar et al., 2018; Wei et al., 2020; Luhach et al., 2021; Zaki et al., 2022; Sadeghi et al., 2024). In addition, accumulating research on MDD and PSD pathogenesis supports the inflammation hypothesis (Beurel et al., 2020; Medeiros et al., 2020; Nagy et al., 2020; Ting et al., 2020; Wu et al., 2020; Hu et al., 2021; Li Z. et al., 2021; Chen et al., 2022; Scaini et al., 2022; Guo et al., 2023; Novakovic et al., 2023; Feng et al., 2024; Wen et al., 2024; Wu et al., 2025). Therefore, the present study explored cilostazol’s PSD development prevention mechanism, focusing on its regulation of neuronal apoptosis and microglial inflammatory signaling pathways.

IS causes varying degrees of brain damage and cognitive impairment (Einstad et al., 2021), and depression is often accompanied by cognitive deficits (Varghese et al., 2022). Central neuronal damage and apoptosis associated with IS, depression, and PSD have been extensively explored (Shao et al., 2020; Wang et al., 2021; Wijeratne and Sales, 2021; Yang et al., 2021; Abdelkawy et al., 2024; Lu et al., 2024; Lei et al., 2025). The inhibition of neuronal damage and apoptosis may be an effective strategy for alleviating cognitive dysfunction in both IS and depression (Wang et al., 2021; Ge et al., 2024; Xu et al., 2024). In the present study, Nissl staining revealed that some hippocampal (a key center for memory and cognition) neurons in the PSD group exhibited disordered arrangement, uneven cytoplasmic staining, and swelling or rupture of certain granular neurons, while, neurons in the cilostazol-treated group exhibited a normal structure. Immunofluorescence co-localization of NeuN and caspase-3, along with differential expression of Bcl2 and Bax upstream of caspase-3, further confirmed that cilostazol significantly reduced hippocampal neuronal apoptosis in CUMS-subjected IS mice. These results (Section 3.2) demonstrate that cilostazol effectively protects hippocampal neurons from damage and apoptosis in PSD model, thereby exerting its PSD-preventive effects.

Following IS, the ischemic and hypoxic environment in the infarcted region activates the microglia (Wei et al., 2024), which release large amounts of pro-inflammatory cytokines, such as TNF-α, IL-1β, and IL-6, exacerbating neuronal damage (Brown and Neher, 2014; Norris and Kipnis, 2019; Hu et al., 2020; Lee et al., 2021; Zheng et al., 2021; Li B. et al., 2022; Kokkosis et al., 2024; Zhang X. et al., 2024). The present study’s results (Section 3.3) revealed an increase in the number and activation of microglia in the PSD mice, suggesting that microglial dysfunction may influence PSD onset and progression. Additionally, patients with PSD often have elevated systemic inflammation (Hu et al., 2021; Li Y. et al., 2022; Wen et al., 2024; Xie et al., 2025). Elevated pro-inflammatory cytokines exacerbate neuronal damage and apoptosis, potentially driving PSD pathogenesis (Feng et al., 2024). To test this premise, we investigated the effects of cilostazol on the inflammation levels in the hippocampal tissue of PSD mice. The study results (Sections 3.3 and 3.4) demonstrated that cilostazol significantly inhibited hippocampal microglial proliferation and activation, reducing TNF-α and IL-1β levels, indicating that cilostazol may have protected hippocampal neurons from damage and apoptosis by regulating microglial activation and pro-inflammatory factors secretion.

Next, we explored the signaling pathways via which cilostazol alleviates hippocampal inflammation in PSD mice. Cilostazol was found to inhibit PDE3 activity, thereby preventing cAMP degradation (Park et al., 2013; Kherallah et al., 2022). Moreover, our exploration of the GEO database found that altered RhoA expression might be closely related with PSD development (Section 3.4). A previously study indicated that increased RhoA expression was associated with decreased exploratory behavior, increased anxiety-like behavior, and decreased dendritic complexity of hippocampal neurons in CUMS-subjected rats (Li et al., 2023). Additionally, cAMP-PKA directly regulates RhoA activation (Aslam et al., 2010; Aburima et al., 2013; Li L. et al., 2021), and the activated form of RhoA (RhoA-GTP) directly influences NF-κB activation, increasing NF-κB p65 levels. In the nucleus, NF-κB p65 acts as a transcription factor, promoting pro-inflammatory cytokine expression (Hayden and Ghosh, 2008; He et al., 2011; Kim et al., 2017; Kim et al., 2018). Furthermore, the cilostazol target proteins, PDE3A and PDE3B, are primarily expressed in microglia, astrocytes, and oligodendrocytes, with low expression in neurons. Notably, PDE3B is highly expressed in microglia (Mitome-Mishima et al., 2013; Bernier et al., 2019; Sadeghi et al., 2024). The inhibition of microglial PDE3B with cilostamide (10 mM) or amrinone (500 mM) was demonstrated to increase cAMP levels in microglia, transforming them into a phenotype enriched in filopodia, dynamically extending and retracting structures essential for the “real-time surveillance” of brain parenchyma (Bernier et al., 2019). When microglia are activated by LPS, cAMP levels decrease, while the anti-inflammatory molecule, adrenomedullin 2, can increase cAMP levels and suppress inflammatory mediators such as TNF-α, IL-1β, COX-2 and iNOS, thereby inhibiting LPS-induced microglial activation (Sun et al., 2021). In high inflammation milieus, pro-inflammatory cytokines exacerbate microglial activation, which is linked to cAMP level changes. For example, TNF treatment reduces the ability of adenylyl cyclase to synthesize cAMP in microglia by up to 50%, and TNF induces the time-dependent degradation of IκBα as well, promoting NF-κB activation. This degradation can be reversed by NF-κB inhibitors such as N-tosyl-L-phenylalanine chloromethyl ketone and N-CBZ-Leu-Leu-Leu-al (Patrizio, 2004). This suggests that NF-κB is involved in the TNF-driven regulation of adenylyl cyclase in the microglia. Furthermore, the accumulation of cAMP in astrocytes was not affected by TNF, although it inhibits the ability of the microglia to synthesize cAMP, which exacerbates their pro-inflammatory response. This inhibition enhances the release of pro-inflammatory and/or cytotoxic factors, leading to neuroinflammation and neuronal damage (Patrizio, 2004). These findings suggest that, post-IS, reduced cAMP levels in microglia impair their ability to regulate RhoA activation and other pathways, leading to aberrant NF-κB signaling, excessive production of pro-inflammatory cytokines, neuronal damage, and ultimately, PSD development. The study results in Sections 3.3 and 3.4 revealed that, PSD mice had enhanced RhoA expression and activation in their hippocampal tissue, accompanied by significantly elevated NF-κB p65, TNF-α, and IL-1β levels, compared with the control mice. Cilostazol significantly reduced RhoA expression and activation and NF-κB p65, TNF-α, and IL-1β levels in PSD mice hippocampal tissues. These findings collectively suggest that cilostazol may alleviate hippocampal inflammation in PSD mice by inhibiting the RhoA/NF-κB signaling pathway in the microglia.

To fully determine the targeted and central roles of the RhoA/NF-κB pathway and microglia, respectively, and to confirm this hypothesis, we further constructed a BV2 microglial cell model with RhoA overexpression and activation, and collected their secretions for primary hippocampal neuron culture. The experimental results revealed that RhoA activation significantly increased NF-κB p65 expression and TNF-α and IL-1β secretions in the BV2 microglial cells (Section 3.5.1). In contrast, cilostazol treatment significantly elevated cAMP levels in the BV2 microglial cells, reduced RhoA-GTP and NF-κB p65 expressions, and decreased TNF-α and IL-1β secretions (Section 3.5.2). Similarly, a previous study demonstrated that RhoA signaling pathway activation led to microglial activation in the trigeminal nucleus caudalis (Jing et al., 2019). Another study compared RhoA expression levels in peripheral blood monocytes from patients with severe IS before and after 90 days of cilostazol and clopidogrel treatment, finding that RhoA expression was significantly suppressed post-treatment (Sheu et al., 2014). Cilostazol reportedly inhibits NF-κB activation by suppressing RhoA activation and downregulating IL-23 production (Park et al., 2013). cAMP can inhibit RhoA signaling activation (Aburima et al., 2013; Fan et al., 2020; Li L. et al., 2021). For example, rhodiola rosea extract inhibits intermittent hypoxia-induced RhoA activation in human umbilical vein endothelial cells by increasing intracellular cAMP levels (Li L. et al., 2021). Glucagon-like peptide one inhibits RhoA expression and reverses cardiac hypertrophy via the cAMP-PKA signaling pathway (Fan et al., 2020). Moreover, cAMP can regulate platelet shape changes via the RhoA-Rho kinase-MLC phosphatase signaling pathway (Aburima et al., 2013). These findings and that of the present study in Sections 3.5.1 and 3.5.2, consistently indicate that cilostazol increases cAMP levels in RhoA-overexpressed BV2 microglial cells, thereby inhibiting RhoA activation-induced microglial activation and pro-inflammatory cytokine secretion.

Furthermore, we cultured primary hippocampal neurons with the conditioned medium from the RhoA-activated BV2 microglia, both with and without cilostazol, to investigate the intervention’s neuroprotective effects. The results (Section 3.5.3) revealed that compared to neurons cultured under normal conditions, those cultured with the conditioned medium exhibited significantly decreased dendritic length and branching, along with a marked increase in apoptotic markers. However, the conditioned medium did not induce neuronal damage. Notably, directly adding cilostazol to the neurons did not reverse the damage caused by the conditioned medium, further confirming that the secreted cytokines from the RhoA-activated microglia primarily contributed to neuronal injury or apoptosis. These findings suggest that the therapeutic mechanism of cilostazol is primarily via the modulation of microglial function to maintain the neuronal growth microenvironment. Collectively, the results (Sections 3.5.1, 3.5.2, and 3.5.3) clearly demonstrated that cilostazol inhibited the excessive secretion TNF-α and IL-1β, via the cAMP/RhoA/NF-κB signaling pathway, thereby preventing neuronal damage and apoptosis.

An intracerebral hemorrhage mouse study reported that cilostazol moderately reduced IBA1 expression in the brain tissue, albeit not leading to a significant decrease in inflammation levels (Sumbria et al., 2017). Although this conclusion is not entirely consistent with our study’s findings, this discrepancy may be attributed to differences in the experimental models and cilostazol’s multifaceted pharmacological effects. Sumbria et al. focused on intracerebral hemorrhage in mice, where blood entering the brain parenchyma post-hemorrhage triggers brain inflammation. Cilostazol’s effects on platelet aggregation inhibition, vasodilation, and increased capillary patency could have promoted bleeding, counteracting its anti-inflammatory effects by inhibiting microglial activation (Sumbria et al., 2017). Conversely, our study focused on IS mice, where the ischemic-hypoxic microenvironment and the resulting cellular debris contribute to brain inflammation. Cilostazol promotes vasodilation in IS mice, facilitating oxygen and nutrient exchange between brain tissue and peripheral blood, and restoring homeostasis while suppressing microglial activation and pro-inflammatory cytokine secretion via the cAMP/RhoA/NF-κB signaling pathway, thereby reducing brain tissue inflammation (Shan et al., 2024).

Overall, our study reaffirms the PSD-preventive effects of cilostazol and is the first to identify a novel mechanism via which cilostazol inhibits microglial activation and alleviates neuronal damage during PSD development. This finding provides clear and compelling evidence for cilostazol’s potential use in the clinical treatment of PSD. Additionally, our study’s notable implications in controlling the abnormal activation of microglia and maintaining brain immune homeostasis, may provide new avenues for brain immune regulation research and the treatment of various neurological disorders.

## 5 Limitations

Although our research clearly demonstrates that cilostazol upregulates cAMP levels in microglia, thereby inhibiting RhoA/NF-κB activation and TNF-α and IL-1β secretion, uncertainties regarding its therapeutic mechanisms and clinical applicability remain. For example, based on the GEO database predictions and existing literature, we identified RhoA as a potential target of cAMP; however, the downstream signaling pathways of cAMP are broad, and the synergistical contribution of other signaling systems remains unclear. Single-cell RNA sequencing and experimental analyses would help further clarify this issue. Additionally, although PDE3, cilostazol’s target, is highly expressed in astrocytes, as reported in the literature, the effects of cilostazol on astrocyte regulation and the underlying mechanisms have not been systematically explored. Furthermore, because the study’s experiments were conducted in mice, using mouse primary cells and cell lines, all data are applicable to these models. Therefore, the specific dosage, treatment duration, and other factors related to cilostazol administration may not be directly translatable to humans with IS, and further small-scale validation studies in different patient populations are needed before large-scale clinical trials on cilostazol for PSD prevention can be initiated. Additionally, some animal experiments used a modest number of biological replicates owing to unavoidable resource constraints. Although this sample size is statistically adequate and all experimental results are consistent, future investigations should incorporate larger cohort sizes to validate and extend our findings.

## 6 Conclusion and outlook

**FIGURE 8:**
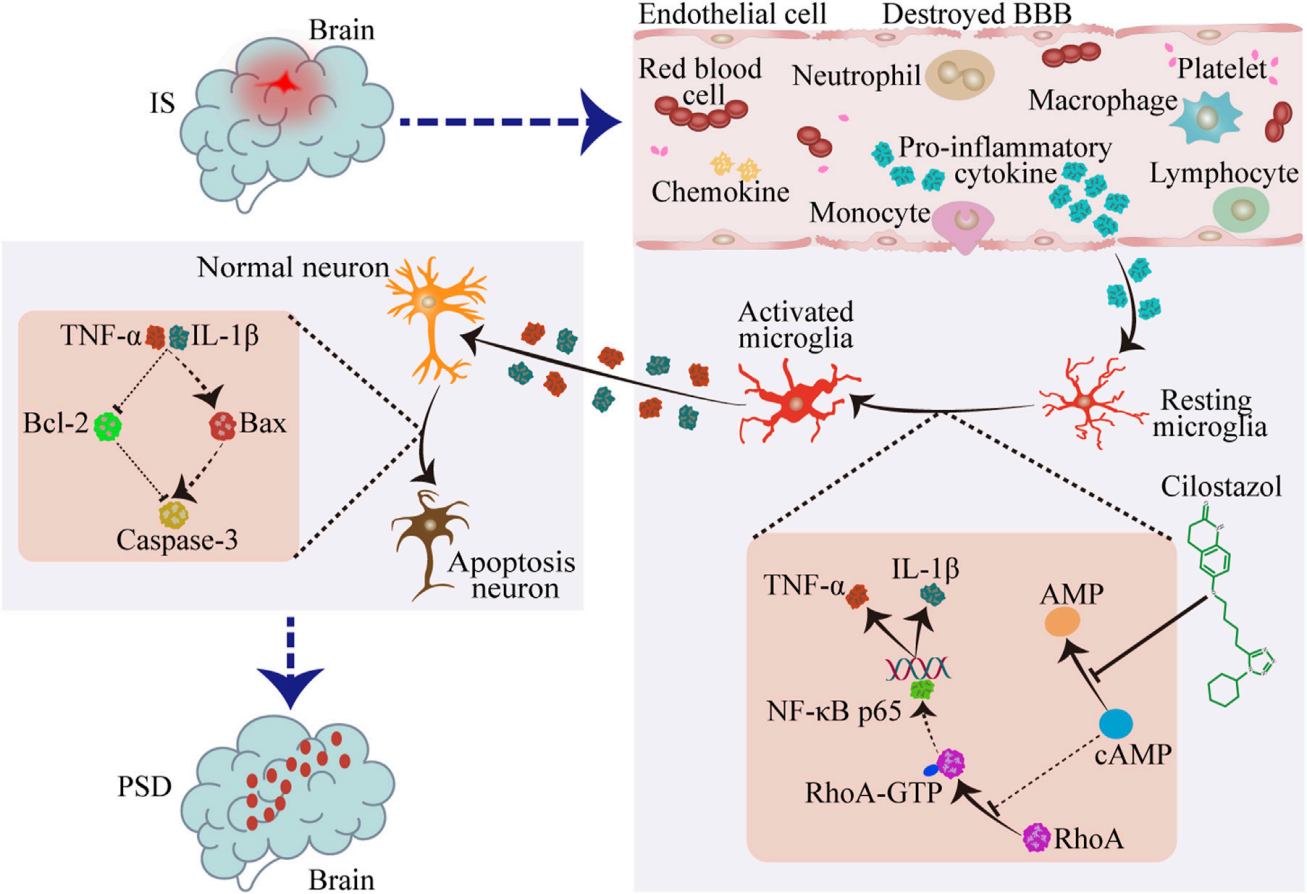
Cilostazol suppresses the RhoA/NF-κB signaling pathway in the microglia to prevent post-stroke depression (PSD). After an ischemic stroke (IS), the blood brain barrier (BBB) is compromised, allowing neutrophils, monocytes, lymphocytes, macrophages, and various pro-inflammatory cytokines and chemokines to infiltrate the brain parenchyma. This infiltration activates the resting microglia, which subsequently produce a considerable amount of pro-inflammatory cytokines, such as TNF-α and IL-1β. The sharp increase in inflammation within the brain tissue microenvironment leads to neuronal apoptosis and dysfunction, ultimately causing PSD as a complication. Cilostazol inhibits the hydrolysis of cAMP, thereby maintaining its suppressive effect on the RhoA/NF-κB signaling pathway. This limits microglial activation, reduces pro-inflammatory cytokine secretion, and provides neuroprotective effects to prevent PSD development. ➝ Promoting effect; ⊣ Inhibitory effect.

Cilostazol, as a PDE3 inhibitor, effectively increases cAMP levels in the hippocampal tissue of PSD mice, strongly inhibits RhoA/NF-κB signaling pathway activation in the microglia, reduces microglial activation, and decreases TNF-α and IL-1β secretion. This in turn lowers the inflammatory levels in the hippocampal tissue, preventing neuronal damage or apoptosis due to high levels of inflammatory signals in the cellular microenvironment (Figure 8). Recent research on cilostazol and the experimental results of this study indicate that cilostazol is a promising new anti-inflammatory and antidepressant drug. It offers clear and unique advantages for the prevention of PSD in patients with IS. Complementing its well-established effects in anti-platelet aggregation and vasodilation, this study provides strong evidence of cilostazol’s PSD prevention potential via its anti-inflammatory mechanisms. This expands cilostazol’s therapeutic use, which is advantageous in accelerating the development of new clinical applications. Nevertheless, further clinical trials are necessary to confirm these findings. The relationship between cilostazol’s anti-inflammatory effects and PSD incidence in patients with IS requires additional data collection and analyses. Furthermore, the cAMP/RhoA/NF-κB signaling pathway was identified as a key mechanism via which cilostazol alleviates microglial activation, clarifying cilostazol’s molecular targets and signaling pathways. These findings lay a theoretical foundation for cilostazol’s use in treating other related diseases. Further studies on the pharmacological effects of cilostazol, or research into its combination with other drugs, could lead to new breakthroughs in the treatment of various neurological disorders.

## Data Availability

The original contributions presented in the study are included in the article/supplementary material, further inquiries can be directed to the corresponding author.

## Abbreviations

PSD, post-stroke depression; MCAO, middle cerebral artery occlusion; IS, ischemic stroke; CUMS, chronic unpredictable mild stress; PDE3, phosphodiesterase 3; NF-κB, nuclear factor-κB; TNF-α, tumor necrosis factor-alpha; IL, interleukin; MDD, major depressive disorder; IκB, inhibitor of κB kinase; LPS, lipopolysaccharide; Con, control group; Cil, cilostazol group; SPT, sucrose preference test; OFT, open-field test; TST, tail suspension test; MWMT, Morris water maze test; HPLC, high-performance liquid chromatography; DMEM, Dulbecco’s Modified Eagle Medium; DMSO, dimethyl sulfoxide; FBS, fetal bovine serum; PBS, phosphate-buffered saline; qPCR, quantitative polymerase chain reaction; ANOVA, analysis of variance.
